# A Systematic Screening of Traditional Chinese Medicine Identifies Two Novel Inhibitors Against the Cytotoxic Aggregation of Amyloid Beta

**DOI:** 10.3389/fphar.2021.637766

**Published:** 2021-04-09

**Authors:** Liang Ma, Jiaojiao Zheng, Huijing Chen, Xia Zeng, Shilin Wang, Chen Yang, Xi Li, Yushuo Xiao, Ling Zheng, Hong Chen, Kun Huang

**Affiliations:** ^1^ Affiliated Wuhan Mental Health Center, Tongji Medical College, Huazhong University of Science and Technology, Wuhan, China; ^2^ Tongji School of Pharmacy, Tongji Medical College, Huazhong University of Science and Technology, Wuhan, China; ^3^ School of Pharmacy, Hubei University of Chinese Medicine, Wuhan, China; ^4^ College of Life Sciences, Wuhan University, Wuhan, China

**Keywords:** Alzheimer’s disease, amyloid β, traditional Chinese medicine, cytotoxicity, sinapic acid, tetrahydroxystilbene-2-O-β-D-glucoside

## Abstract

The toxic aggregates of amyloid beta (Aβ) disrupt the cell membrane, induce oxidative stress and mitochondrial dysfunction, and eventually lead to Alzheimer’s disease (AD). Intervening with this cytotoxic aggregation process has been suggested as a potential therapeutic approach for AD and other protein misfolding diseases. Traditional Chinese Medicine (TCM) has been used to treat AD and related cognitive impairment for centuries with obvious efficacy. Extracts or active ingredients of TCMs have been reported to inhibit the aggregation and cytotoxicity of Aβ. However, there is a lack of systematic research on the anti-Aβ aggregation effects of TCM components. In this study, we performed a systematic screening to identify the active ingredients of TCM against the cytotoxic aggregation of Aβ42. Through a literature and database survey, we selected 19 TCM herbals frequently used in the treatment of AD, from which 76 major active chemicals without known anti-amyloid effects were further screened. This took place through two rounds of MTT-based screening detection of the cytotoxicity of these chemicals and their effects on Aβ42-induced cytotoxicity, respectively. Tetrahydroxystilbene-2-O-β-D-glucoside (TSG) and sinapic acid (SA) were found to be less toxic, and they inhibited the cytotoxicity of Aβ42. Further studies demonstrated that TSG and SA concentration-dependently attenuated the amyloidosis and membrane disruption ability of Aβ42. Thus, we identified two novel chemicals (TSG and SA) against the cytotoxic aggregation of Aβ42. Nonetheless, further exploration of their therapeutic potential is warranted.

## Introduction

Alzheimer’s disease (AD) is a common age-associated neurodegenerative disease, characterized by progressive memory and neuronal loss combined with cognitive impairment (Hung and Fu, 2017). Pathologically, one of the major features of AD brain is the deposition of amyloid plaques, mostly comprising insoluble fibrillar amyloid-β (Aβ) (Chiti and Dobson, 2017; Soto and Pritzkow, 2018) including Aβ40 and Aβ42 (Hardy and Allsop, 1991; Ghiso and Frangione, 2002). Compared to Aβ40, Aβ42 is more prone to fiberise owing to its more hydrophobic nature. Therefore, it is commonly used to study the role of protein aggregation in the onset and progression of AD (Findeis, 2007; Qiu et al., 2015).

Aggregates of Aβ42 have been reported to cause cell damage through different cytotoxic mechanisms such as permeabilisation of the lipid membrane, induction of oxidative stress and mitochondrial dysfunction, and impairment of the cellular protein degradation system, which may eventually result in AD (Aguzzi and O'Connor, 2010; Lee et al., 2017; Chen et al., 2019; Xiao et al., 2019). Inhibiting the generation of toxic aggregates of amyloid proteins with antibodies, peptide/protein mimics, or small molecules have been suggested as therapeutic approaches for AD or other protein misfolding diseases (Li et al., 2012; Cheng et al., 2013; Wang et al., 2019; Ma et al., 2020). For example, we and others have previously reported that food-derived natural chemicals, such as (-)-epigallocatechin 3-gallate (EGCG), tanshinone, curcumin, magnolol, myricetin, caffeic acid, and proanthocyanidins showed inhibitory effects on amyloid formation and related cytotoxicity (Cheng et al., 2011; Jiao et al., 2013; Gong et al., 2014; Guo et al., 2015; Ji et al., 2016; Zhang et al., 2018).

Traditional Chinese Medicine (TCM) has been used to treat AD and related cognitive impairment for centuries (Su et al., 2014; Pei et al., 2020). Extracts or active ingredients of TCMs show anti-AD effects via multiple mechanisms, such as improving acetylcholine levels and decreasing acetyl cholinesterase activity (Wang et al., 2018), suppressing abnormal phosphorylation of Tau protein (Ma et al., 2019), inhibiting neuronal apoptosis (Wang et al., 2019; Zhang et al., 2019), anti-oxidation (Tong et al., 2018), anti-inflammation (Maione et al., 2018), and inhibiting Aβ42 deposition (Shi et al., 2018; Zhang et al., 2019). Moreover, extracts or active ingredients of TCMs can inhibit the aggregation and cytotoxicity of Aβ (Shin et al., 2018; Wang et al., 2020). However, there are a lack of systematic reports on the anti-Aβ aggregation effects of TCM chemicals.

In this study, we performed a systematic investigation to identify the active ingredients of TCM against the cytotoxic aggregation of Aβ42. First, we selected the 19 most commonly used TCMs in treating AD and related cognitive impairments through literature investigation (Yan, 2007; Yan and Chen, 2008; Hu et al., 2012; Howes et al., 2017; Lin et al., 2019), from which 76 chemicals were selected through composition investigation. An MTT-based cytotoxicity assay and thioflavin-T (ThT) fluorescence assay were performed to screen chemicals that simultaneously inhibit the cytotoxicity and aggregation of Aβ42. Transmission electron microscopy (TEM), dynamic light scattering (DLS) analysis and dye leakage assays were further performed to investigate the effects of these chemicals on the aggregation and membrane disruption ability of Aβ42.

## Materials and Methods

### Materials

Synthetic amyloid-β (1–42) was obtained from LifeTein (Beijing, China). All chemicals were obtained from Shanghai Aladdin Biochemical Technology Co., Ltd (Shanghai, China) and Yuanye Biotech (Shanghai, China) and of the highest grade available. ThT and 5(6)-carboxyfluorescein were obtained from Sigma–Aldrich (St. Louis, MO). Coagulation reagent I (DOPE: DOPS: DOPC 5:3:2 w/w) was obtained from Avanti Polar Lipids (Alabaster, AL).

### Chemical Selection and Screening

Literature investigation was conducted using the largest Chinese academic literature database, China National Knowledge Infrastructure (CNKI). Twenty articles were identified by using the keyword “dementia” and “medication rule”. From these articles, the 19 most frequently used TCMs in the treatment of AD were selected, and the 10 top-reported active chemicals for each of the 19 TCMs were selected through literature investigation; finally, and 190 chemicals were identified. Among these 190 chemicals, we excluded 114, which were not commercially available or had been reported to affect protein aggregation. The remaining 76 chemicals were further experimentally screened for their cytotoxicity and anti-aggregation properties.

### MTT Assay

An MTT-based toxicity assay was performed as we previously described (Ma et al., 2018). SH-SY5Y cells were cultured in Dulbecco’s modified Eagle medium with high glucose medium containing 8% foetal bovine serum, 1% penicillin–streptomycin solution and 1% sodium pyruvate. The cells were plated in 96-well plates at a density of 3 × 10^3^ cells/well and cultured for 24 h. After the addition of fresh medium containing different concentrations of chemicals and/or 20 μM Aβ42 (20 μM chemicals in the initial screening for cytotoxicity of chemicals, and 20/200 μM chemicals to measure their effects against Aβ42-induced cytotoxicity), the cells were incubated for another 24 h. Then, 10 μl of MTT (5 mg/ml) was added into each well, followed by the addition of 100 μL dimethyl sulfoxide (DMSO). The absorbance was measured at 570 nm.

### Amyloid Formation and ThT Fluorescence Assays

Formation and disaggregation of Aβ42 fibrils were monitored via ThT fluorescence detection. For amyloid formation, Aβ42 was dissolved in 60 mM NaOH to a stock concentration of 200 μΜ and then diluted in 50 mM phosphate-buffered saline (PBS) (100 mM NaCl, pH 7.4) to a final concentration of 20 μΜ as we previously reported (Zhang et al., 2018). Chemicals TSG, SA and EGCG were dissolved in DMSO to prepare stock solutions, which were mixed with 20 μM Aβ42 at the indicated molar ratios (1:1 or 10:1). The samples were incubated at 37°C for amyloid formation. At the designated time points, 10 μl aliquots were mixed with ThT (final concentration of 20 μM) and fluorescence was measured using an FL-2700 fluorescence spectrophotometer (Hitachi, Tokyo, Japan) with excitation and emission wavelengths set at 450 and 482 nm, respectively. For disaggregation experiments, preformed fibrils were generated by incubating 20 μM Aβ42 for 6 h; then, TSG, SA, or EGCG was mixed with preformed fibrils at the indicated molar ratios (1:1 or 10:1), and designated time points. Next, 10 μL aliquots were mixed with ThT and the fluorescence intensity was detected. All experimental groups were in triplicate and all experiments were repeated at least three times.

### Transmission Electron Microscopy

The samples for TEM were prepared as we previously described (Ma et al., 2018). Briefly, 5 μl of sample was pipetted onto a 400-mesh Formvar/carbon coated copper grid (Zhongjingkeyi Tech., Beijing, China) and incubated for 5 min at 25°C. After removing excess solvent, 1% (m/v) freshly prepared uranyl acetate was added dropwise for negative staining and then examined using a transmission electron microscope (Hitachi, Tokyo, Japan) operating at an accelerating voltage of 120 kV.

### Dynamic Light Scattering Analysis

DLS was performed using a ZetaPals potential analyzer (Brookhaven Instruments, New York, NY) as we described previously (Guo et al., 2015). Briefly, the samples were measured with a scattering angle of 90°. Each sample was scanned three times (30 s per scan) and the mean particle size and multimodal size distribution were recorded.

### Dye Leakage Assays

Dye leakage assays were performed as we previously described (Ma et al., 2018). Briefly, coagulation reagent I was dissolved in chloroform, dried with N_2_ into a lipid film and lyophilised for 12 h. Carboxyfluorescein was dissolved in 50 mM PBS to a final concentration of 40 mM and then added onto the lipid films to form vesicles. Vesicles containing carboxyfluorescein were further purified using a PD-10 column (Sangon Biotech., Shanghai, China). Samples of Aβ42 were added to coagulation reagent I vesicles at a final concentration of 1 μM and incubated at 37°C for 10 min to induce membrane disruption. Then, the fluorescence intensity was measured at excitation and emission wavelengths of 493 and 518 nm, respectively. Vesicles treated with 0.2% Triton X-100, which completely disrupts the membrane, was used as the control. All experiments were repeated at least three times.

### Statistical Analysis

Each experiment was repeated at least three times and the data were expressed as the average ± standard deviation. The Kruskal–Wallis test and the Mann–Whitney test were used to evaluate statistical significance. Differences were considered statistically significant at *p* < 0.05.

## Results

### Screening Process

Through investigating the largest Chinese academic literature database CNKI using the keyword “dementia”, 223,224 results were obtained (as of 12/31/2019). Then, after further screening using “medication rule”, 55 publications were obtained (as of 12/31/2019). Fifteen of these 55 publications focused on vascular dementia, eight on schizophrenia, 12 on TCM theory against dementia, and 20 on the medication rules of TCM in AD treatment.

We further identified the 10 most frequently used AD-treating TCMs summarized in these 20 articles (Supplementary Table S1), and a total of 24 TCMs were found, of which 19 appeared at least twice (Supplementary Table S2). As *Cinnabar*, which appeared twice, mainly comprised toxic mercury sulphide, we replaced it with *Ginkgo biloba* which appeared once but has been reported to be effective in clinical trials against AD (Savaskan et al., 2018; Liu et al., 2019). A PUBMED search revealed that all these 19 TCMs have been reported to be beneficial in AD animal or cellular models (Supplementary Table S2).

We further investigated these articles on CNKI to identify the 10 top-reported active chemicals of each TCM, and a total of 190 chemicals were selected. From the 116 commercially available chemicals among these 190 chemicals (Supplementary Table S3), literature studies suggested 40 of them had been reported to show different effects on protein aggregation (Supplementary Table S3). Therefore, we performed experimental screening on the remaining 76 chemicals.

### Experimental Screening of Chemicals That Inhibit the Cytotoxicity of Aβ42

**FIGURE 1:**
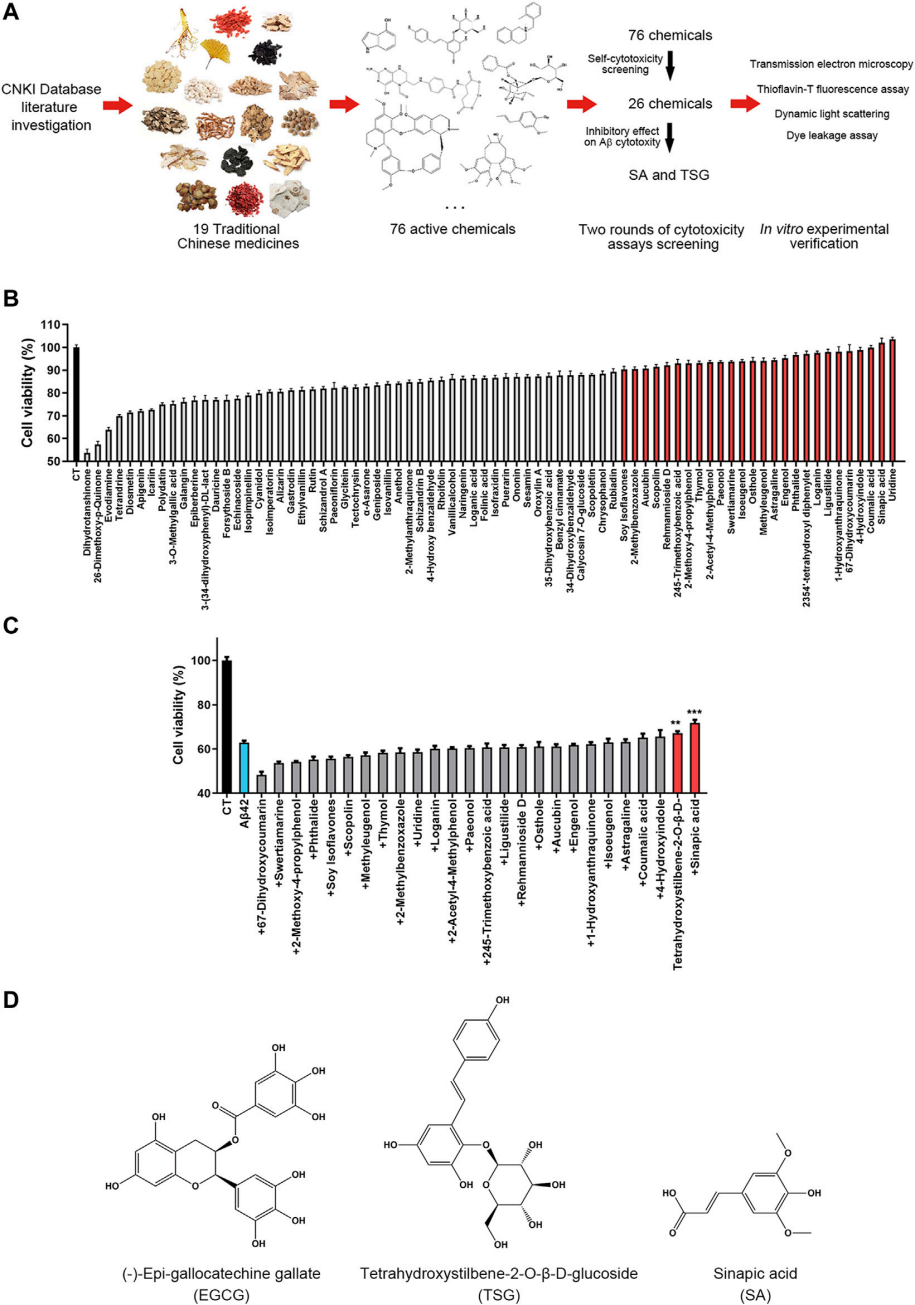
Experimental design and chemical screening. **(A)** The experimental design of this article; **(B)** Screening the less toxic chemicals from all 76 chemicals, the chemicals with minor cytotoxicity (<10%) were marked red; **(C)** Screening the chemicals that significantly inhibit the cytotoxicity of Aβ42, the chemicals that inhibit the cytotoxicity were marked red, **p < 0.01, ***p < 0.001; **(D)** The structure of (-)-epi-gallocatechine gallate (EGCG), Tetrahydroxystilbene-2-O-β-D-glucoside (TSG) and sinapic acid (SA).

The screening process involved two steps; first, the cytotoxicity of 76 chemicals was measured; and second, the effects of these chemicals on Aβ42-induced cytotoxicity were determined (Figure 1A). In an MTT-based cytotoxicity assay, we first detected the cytotoxicity of 76 chemicals at a concentration of 20 μM. Seventeen of these 76 chemicals induced more than 20% cell death, and 33 chemicals showed cytotoxicity between 10 and 20%; 26 chemicals exhibiting cytotoxicity below 10% were further studied (Figure 1B and Supplementary Table S4). The effects of these 26 chemicals on the cytotoxicity of Aβ42 in SH-SY5Y cells were investigated; 20 μM Aβ42 induced a high cell death rate (36.4 ± 1.30%), twenty-four chemicals significantly enhanced toxicity or showed no obvious effects when co-incubated with Aβ42 (Figure 1C and Supplementary Table S4). Two chemicals, tetrahydroxystilbene-2-O-β-D-glucoside (TSG, Figure 1D) and sinapic acid (SA, Figure 1D), significantly attenuated the cytotoxicity of Aβ42 by 4.1 ± 2.4% and 8.8 ± 3.6%, respectively (*p* < 0.05) (Figure 1C and Supplementary Table S4).

### Chemicals Tetrahydroxystilbene-2-O-β-D-Glucoside and Sinapic Acid Concentration-dependently Inhibit the Cytotoxicity and Aggregation of Aβ42

**FIGURE 2:**
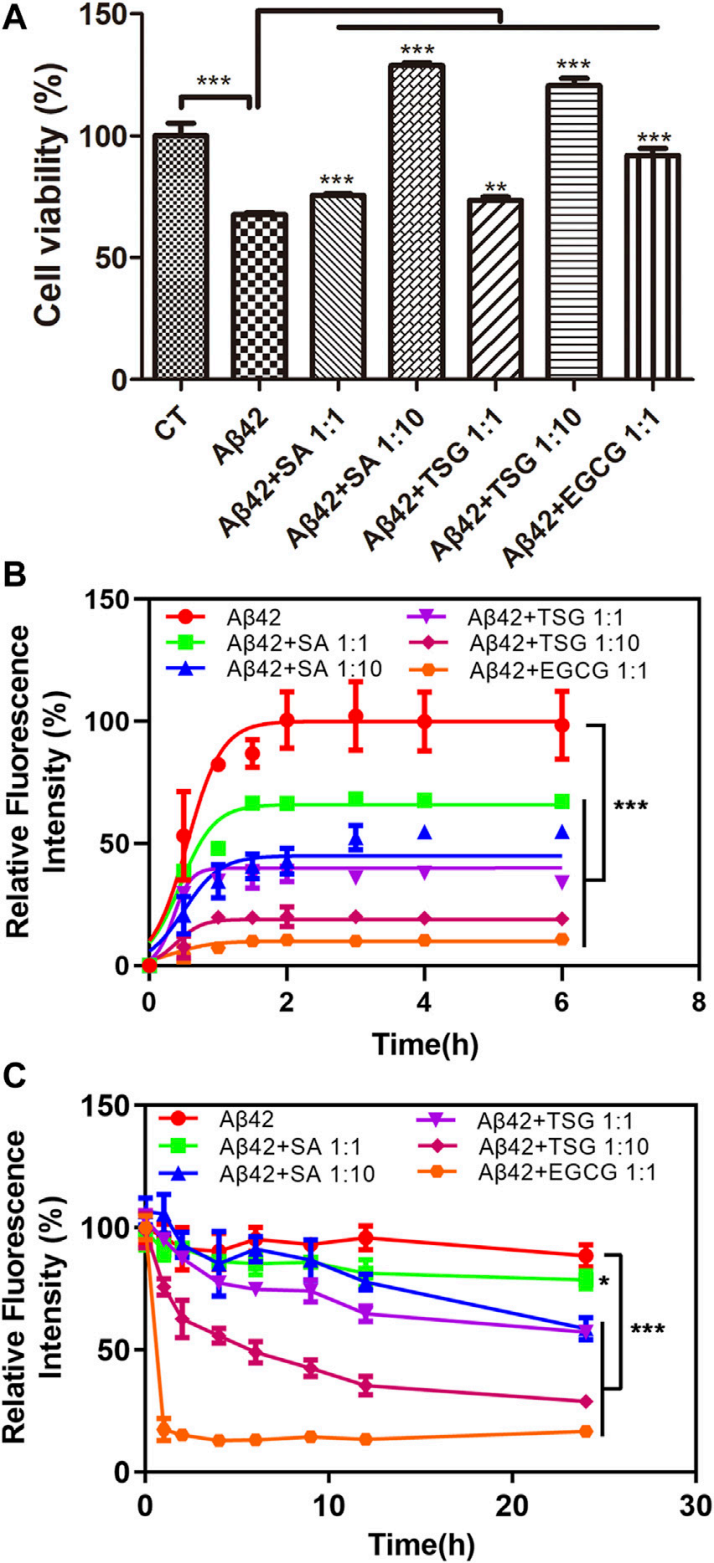
Effects of equimolar EGCG, SA, TSG, and 10-fold SA, TSG on the cytotoxicity and aggregation of Aβ42. **(A)** The effects of equimolar and 10-fold SA and TSG on Aβ42 cytotoxicity; **(B)** The effects of equimolar EGCG, SA, TSG and 10-fold SA, TSG on the aggregation of Aβ42; **(C)** The effects of equimolar EGCG, SA, TSG and 10-fold SA, TSG on the disaggregation of Aβ42 fibrils. **p < 0.01, ***p < 0.001.

Epigallocatechin gallate (EGCG), a polyphenol potently inhibiting the aggregation and neurotoxicity of Aβ42 *in vitro* and *in vivo* (Syarifah-Noratiqah et al., 2018; Kim et al., 2019), was used as a positive control to investigate the effects of SA and TSG on the cytotoxicity and aggregation of Aβ42. In the MTT assay, 20 μM Aβ42 induced 30.03 ± 3.82% of cell death. The presence of equimolar EGCG significantly decreased cell death to 8.02 ± 7.07%. SA or TSG at a concentration of 20 μM decreased cell death to 24.47 ± 1.67% and 26.40 ± 3.61%, respectively (Figure 2A).

A ThT-based fluorescence assay was used to measure amyloid formation by Aβ42. Aβ42 exhibited a short lag time of 0.03 ± 0.04 h and approached an aggregation plateau within 2 h (Table 1). The presence of EGCG prolonged the lag time to 0.10 ± 0.05 h and decreased the maximum fluorescence intensity by 89% (Table 1). Both SA and TSG inhibited Aβ42 amyloid formation in a concentration-dependent manner (Figure 2B). The presence of equimolar SA significantly decreased the maximum fluorescence intensity by 33%, while the presence of 10-fold SA (200 μM) extended the lag time of amyloid formation to 0.15 ± 0.03 h and decreased the maximum fluorescence intensity by 51% (Table 1). The presence of equimolar or 10-fold TSG decreased the maximum fluorescence intensity by 64 and 80% and prolonged the lag time to 0.15 ± 0.01 h and 0.36 ± 0.10 h, respectively (Table 1). Moreover, the presence of EGCG, TSG and SA effectively disaggregated the preformed Aβ42 fibrils (Figure 2C).

**TABLE 1:** Effects of SA, TSG and EGCG on the amyloidogenicity (T_50_, lag lime and maximum fluorescence intensity) and cytotoxicity of Aβ42.

	Maximum intensity	T_50_	Lag time	Cytotoxicity
Aβ42	1.00 ± 0.12^a^	0.37 ± 0.06 h	0.03 ± 0.04 h	30.03 ± 3.82%
Aβ42 + SA 1:1	0.67 ± 0.01**^b^	0.63 ± 0.08 h**	0.01 ± 0.02 h^ns^	24.47 ± 1.67%**
Aβ42 + SA 1:10	0.49 ± 0.03**	0.95 ± 0.23 h*	0.15 ± 0.03 h*	−28.87 ± 2.59%***
Aβ42 + TSG 1:1	0.36 ± 0.01***	0.34 ± 0.04 h^ns^	0.15 ± 0.01 h*	26.40 ± 3.61%**
Aβ42 + TSG 1:10	0.20 ± 0.01***	0.62 ± 0.12 h*	0.36 ± 0.10 h**	−20.53 ± 7.56%***
Aβ42 + EGCG 1:1	0.11 ± 0.02***	0.40 ± 0.05 h*	0.10 ± 0.05 h*	8.02 ± 7.07%***

^a^ The maximum fluorescence intensity of Aβ42 was set as 1.

^b^ ^ns^ *p* > 0.05, *p* < 0.05, *p* < 0.01, *p* < 0.001.

### Chemicals Tetrahydroxystilbene-2-O-β-D-Glucoside and Sinapic Acid Inhibit the Formation of Large Fibril Aggregates During Aβ42 Incubation

**FIGURE 3:**
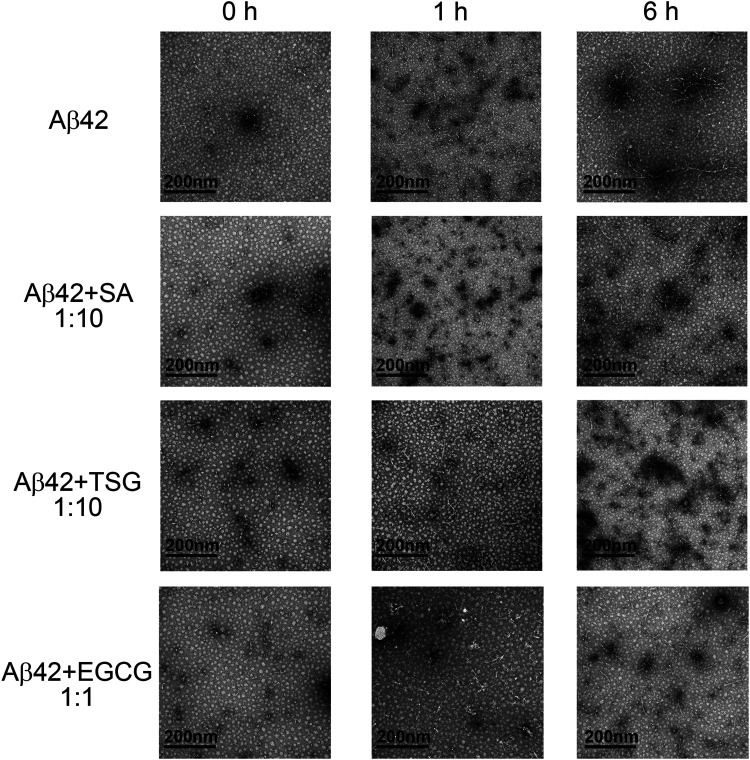
Effects of EGCG, SA and TSG on the morphology of Aβ42 aggregates at different incubation time points.

The morphology of Aβ42 aggregates in the absence or presence of chemicals was assessed by TEM. At the beginning of incubation, samples of all three groups mainly showed punctiform structures (Figure 3). After 1 h incubation, Aβ42 group exhibited long linear structures, indicating the formation of amyloid fibrils, together with a few punctiform structures. The groups with equimolar EGCG and 10-fold SA/TSG still showed punctiform structures after 1 h incubation (Figure 3). When incubated for 6 h, samples of Aβ42 group showed more long ribbon-like fibrils and fewer punctiform structures. Samples from groups with EGCG, SA, and TSG also exhibited short linear fibrils, with punctiform structures remained (Figure 3).

**FIGURE 4:**
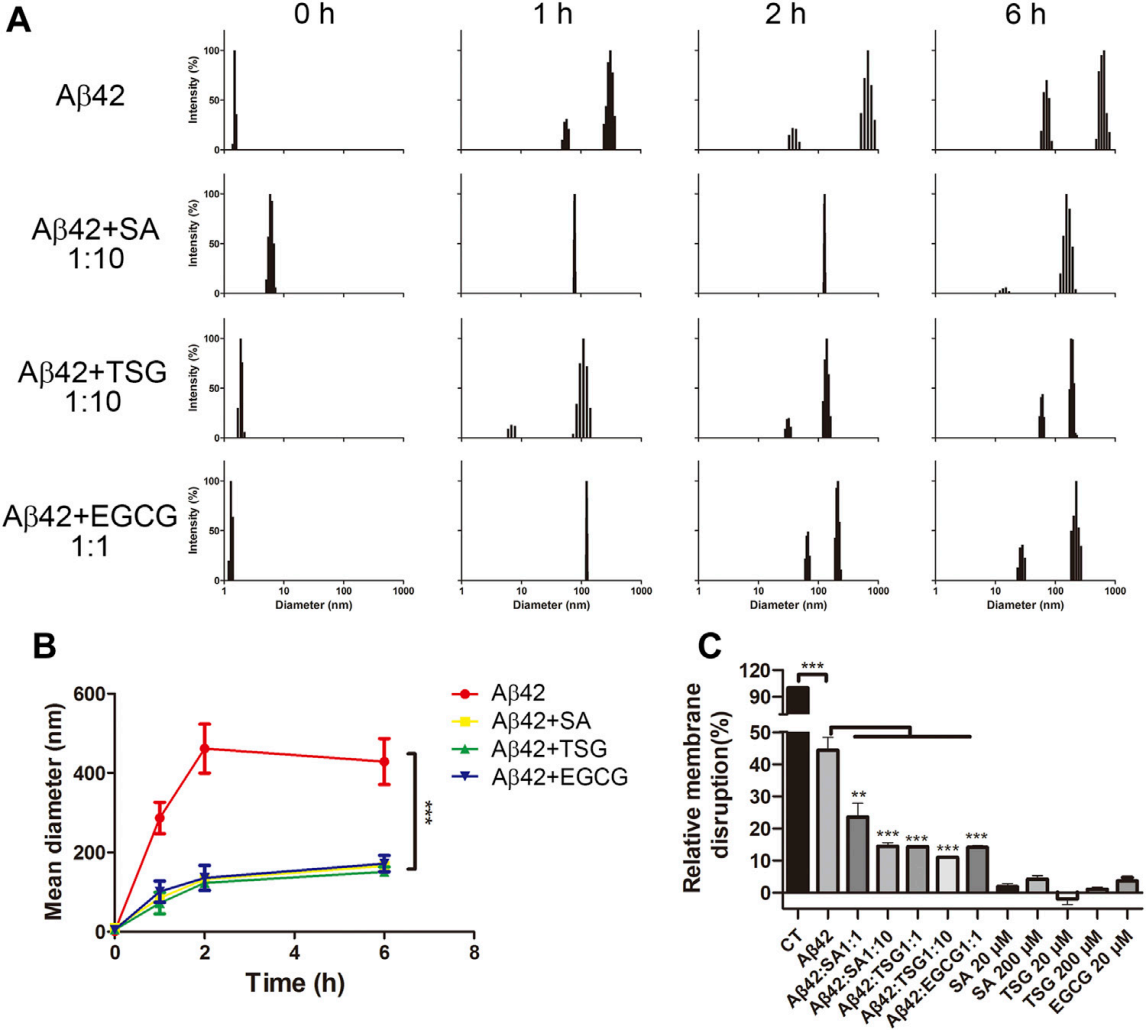
Effects of EGCG, SA and TSG on the partial size distribution during Aβ42 aggregation and on membrane disruption caused by Aβ42 aggregates. **(A)** Dynamic light scattering analysis of different groups; **(B)** Mean diameter changes of different groups; **(C)** Effects of EGCG, SA and TSG on membrane disruption caused by Aβ42 aggregates. **p < 0.01, ***p < 0.001.

To monitor the particle size and distribution during aggregation, DLS assay was performed. The average diameter of all groups was less than 10 nm at the beginning of incubation (Figure 4A). After 1 h incubation, Aβ42 showed particles large than 300 nm, whereas the groups with the EGCG, TSG and SA showed no particles larger than 150 nm (Figure 4A), which was consistent with the TEM results (Figure 3). The average diameter of Aβ42 group was greater than 200 nm after 1 h incubation, whereas that in the EGCG-, TSG- and SA-treated groups was below 200 nm throughout the incubation (Figure 4B).

### Chemicals Tetrahydroxystilbene-2-O-β-D-Glucoside and Sinapic Acid Inhibit Aβ42-Induced Membrane Disruption

Protein aggregates often exhibit cytotoxicity through membrane disruption (Zhang et al., 2018). A dye leakage assay was performed to explore the membrane disruptions. EGCG, SA, and TSG barely impaired (less than 5%) carboxyfluorescein-containing lipid vesicles. The presence of 1 μM Aβ42 destroyed 44.39 ± 4.02% lipid vesicles (Figure 4C). The presence of EGCG significantly decreased the membrane disruption rate to 14.13 ± 0.61%, while the presence of equimolar TSG or SA decreased the membrane disruption rate to 14.33 ± 0.35% and 23.57 ± 4.32%, respectively; 10-fold TSG and SA further decreased the membrane disruption rates to 11.00 ± 0.36% and 14.47 ± 1.10%, respectively (Figure 4C).

## Discussion

For centuries, TCMs have been clinically used in the prevention and treatment of AD and related cognitive impairment with obvious efficacy (Li et al., 2006; Li et al., 2015; Ong et al., 2018). They are mainly used to treat memory decline in the elderly (Howes et al., 2017; Yang et al., 2017; Ong et al., 2018). In the perspective of modern medicine, TCM extracts and their active ingredients have been proved to exhibit anti-AD effects through improving acetylcholine level and decreasing acetyl cholinesterase activity (Wang et al., 2018), suppressing abnormal phosphorylation of Tau protein (Ma et al., 2015), inhibiting neuronal apoptosis (Ji et al., 2020), anti-oxidation (Xu et al., 2017), anti-inflammation (Liu et al., 2014), and inhibiting Aβ deposition (Chiroma et al., 2019) in different studies. Because the toxic aggregation of Aβ may occur in the early stages of AD and induce further apoptosis, inflammation, and neurodegeneration, inhibiting the generation or aggregation of Aβ has been regarded as the preferred therapeutic approach for AD (Takahashi and Mihara, 2008; Liu et al., 2017; Xiong et al., 2017).

Numerous studies have indicated that TCM extracts or active ingredients could inhibit the aggregation and cytotoxicity of Aβ (Shin et al., 2018; Wang et al., 2020). However, there is a lack of systematic investigations on the anti-Aβ aggregation effects of TCM components. In this study, through a literature/database survey, we screened 76 major active chemicals without known anti-amyloid effects from 19 TCM herbals that were frequently used against AD and identified TSG and SA as two novel inhibitors against protein misfolding.

As one of the main active components from *Polygoni Multiflori Radix*, TSG has been reported to have strong anti-inflammatory, antioxidant, anti-apoptotic, and free radical scavenging activities (Wu et al., 2017; Zhang and Chen, 2018; Qian et al., 2020). Extensively found in spices, citrus, berry fruits, and vegetables (Chen, 2016), SA is also known to exhibit antioxidant, anti-inflammatory, anticancer, and neuroprotective activities (Chen, 2016; Zych et al., 2019). Both TSG and SA show protective effects on learning/memory deficits and cognitive impairment in AD animal models (Lee et al., 2012; Sheng et al., 2016; Shahmohamady et al., 2018). However, to our knowledge, there is no report on the anti-Aβ42 aggregation effects of TSG and SA.

In this study, we demonstrated that TSG and SA concentration-dependently inhibited the cytotoxicity of Aβ42, as well as the formation of large fibril aggregates during Aβ42 incubation. In addition, we also found that TSG and SA concentration-dependently inhibited the membrane disruption ability of Aβ42. These results indicated that TSG and SA may reduce the cytotoxicity of Aβ42 through inhibiting the formation of highly ordered aggregates which have strong membrane disruption ability. We also observed a common structural feature in TSG and SA, both contained a benzene ring with a phenolic hydroxyl group whose para position directly connects a carbon–carbon double bond (Figure 1C). Further binding studies are warranted to determine whether the P-vinylphenol group is involved in the interaction between TSG/SA and Aβ42.

In conclusion, we screened 76 major active chemicals without known anti-amyloid effects from 19 TCM herbals frequently used against AD through literature/database survey, and two chemicals TSG and SA were found to concentration-dependently attenuate the amyloidosis and membrane disruption ability of Aβ42. Studies also revealed that TCM contains multiple anti-Aβ toxic aggregation chemicals, such as protocatechuic acid (Hornedo-Ortega et al., 2016), salvianolic acid B (Tang and Zhang, 2001) and tanshinone (Wang et al., 2013) found in *Salviae Miltiorrhizae Radix et Rhizoma*; baicalein (Lu et al., 2011), kaempferol (Hanaki et al., 2016) and isorhamnetin (Iida et al., 2015) in *Astragali Radix* have been reported to inhibit Aβ aggregation (Supplementary Table S3). These results suggest that the anti-AD effects of TCM may be attributed to the synergistic actions of multiple chemicals. Further studies focusing on such synergistic effects are warranted.

## Data Availability

The raw data supporting the conclusions of this article will be made available by the authors, without undue reservation, to any qualified researcher.
