# Supplementary material for: A Systematic Screening of Traditional Chinese Medicine Identifies Two Novel Inhibitors Against the Cytotoxic Aggregation of Amyloid Beta
Source: Front Pharmacol. 2021 Apr 9;12:637766. doi: 10.3389/fphar.2021.637766 (PMC8062920; doi:10.3389/fphar.2021.637766)
Supplement: Supplementary file 1 [file datasheet1.docx]

**Table S1. Literature investigation on the medication rules of TCM in the treatment of Alzheimer's disease and the most frequently used 10 TCMs in each article.** Through investigation the largest Chinese academic database CNKI with the keyword “dementia” and “medication rule”, 55 publications were found. 20 of the 55 publications were focused on exploring the medication rules of TMC in AD treatment. The top 10 anti-AD TCM prescriptions provided in these 20 articles were listed.

| **Literature** | **Most Frequently Used 10 Traditional Chinese medicines** |
| --- | --- |
| Identification the rules of compound traditional Chinese medicine patent treating Alzheimer's disease based on sooPAT search engine (Fan et al., 2019) | *Polygalae Radix, Astragali Radix, Acori Tatarinowii Rhizoma, Chuanxiong Rhizoma, Ginseng Radix et Rhizoma, Salviae Miltiorrhizae Radix et Rhizoma, Poria, Lycii Fructus, Angelicae Sinensis Radix, Rehmanniae Radix* |
| Studies on the medication rules of traditional Chinese medicine for Alzheimer 's disease based on association rules (Zhu, 2017) | *Acori Tatarinowii Rhizoma, Rehmanniae Radix, Chuanxiong Rhizoma, Polygalae Radix, Glycyrrhizae Radix et Rhizoma, Poria, Astragali Radix, Salviae Miltiorrhizae Radix et Rhizoma, Corni Fructus, Angelicae Sinensis Radix* |
| Researches on the medication rules of traditional Chinese medicine in the treatment of Alzheimer 's disease based on association rules (Zeng et al., 2015) | *Acori Tatarinowii Rhizoma, Polygalae Radix, Salviae Miltiorrhizae Radix et Rhizoma, Poria, Glycyrrhizae Radix et Rhizoma, Chuanxiong Rhizoma, Pinelliae Rhizoma, Curcumae Rhizoma, Atractylodis Macrocephalae Rhizoma, Angelicae Sinensis Radix* |
| Studies on the medication rules of Alzheimer 's disease based on cluster analysis (Wang et al., 2012) | *Acori Tatarinowii Rhizoma, Poria, Rehmanniae Radix, Polygalae Radix, Corni Fructus, Angelicae Sinensis Radix, Ginkgo biloba, Lycii Fructus, Chuanxiong Rhizoma, Dioscoreae Rhizoma* |
| Researches on the rules of Alzheimer's disease treatment based on data mining (Jiao, 2016) | *Acori Tatarinowii Rhizoma, Astragali Radix, Poria, Rehmanniae Radix, Glycyrrhizae Radix et Rhizoma, Chuanxiong Rhizoma, Polygalae Radix, Angelicae Sinensis Radix, Salviae Miltiorrhizae Radix et Rhizoma, Lycii Fructus* |
| Analysis on medication rules of treating dementia-related diseases by ancient physicians based on data mining (Yi et al., 2018) | *Poria, Polygalae Radix, Ginseng Radix et Rhizoma, Acori Tatarinowii Rhizoma, Glycyrrhizae Radix et Rhizoma, Angelicae Sinensis Radix, Ophiopogonis Radix, Atractylodis Macrocephalae Rhizoma, Rehmanniae Radix, Os Draconis* |
| Researches on the compatibility of traditional Chinese medicine formulas for treating Alzheimer's disease based on data mining (Lin et al., 2015) | *Ginseng Radix et Rhizoma, Polygalae Radix, Glycyrrhizae Radix et Rhizoma, Angelicae Sinensis Radix, Poria cum Radix Pini, Cinnabaris, Poria, Acori Tatarinowii Rhizoma, Atractylodis Macrocephalae Rhizoma, Rehmanniae Radix* |
| Studies on medication rules of modern Chinese medicine in the treatment of  Alzheimer’s disease based on data mining (Song et al., 2019) | *Acori Tatarinowii Rhizoma, Astragali Radix, Rehmanniae Radix, Salviae Miltiorrhizae Radix et Rhizoma, Poria, Polygalae Radix, Chuanxiong Rhizoma, Polygoni Multiflori Radix, Angelicae Sinensis Radix, Lycii Fructus* |
| Data mining-based analysis on the regularity of ancient Chinese medicine prescription for amnestic mild cognitive impairment (Qu, 2015) | *Ginseng Radix et Rhizoma, Polygalae Radix, Poria, Glycyrrhizae Radix et Rhizoma, Arisaematis Rhizoma, Poria cum Radix Pini, Angelicae Sinensis Radix, Ophiopogonis Radix, Rehmanniae Radix, Acori Tatarinowii Rhizoma* |
| Researches on rules of traditional Chinese medicine in treating Alzheimer's disease based on data mining (Lu et al.) | *Acori Tatarinowii Rhizoma, Chuanxiong Rhizoma, Astragali Radix, Rehmanniae Radix, Polygalae Radix, Poria, Angelicae Sinensis Radix, Salviae Miltiorrhizae Radix et Rhizoma, Corni Fructus, Lycii Fructus* |
| Analysis of medication rules for the treatment of dementia based on the traditional Chinese medicine inheritance auxiliary system (Huang et al., 2019) | *Acori Tatarinowii Rhizoma, Poria, Polygalae Radix, Astragali Radix, Chuanxiong Rhizoma, Rehmanniae Radix, Ginseng Radix et Rhizoma, Glycyrrhizae Radix et Rhizoma, Angelicae Sinensis Radix, Polygoni Multiflori Radix* |
| Researches on traditional Chinese medicine formulas for treating Alzheimer's disease based on traditional Chinese medicine inheritance auxiliary systems(Zong et al., 2014) | *Acori Tatarinowii Rhizoma, Chuanxiong Rhizoma, Polygoni Multiflori Radix, Salviae Miltiorrhizae Radix et Rhizoma, Angelicae Sinensis Radix, Ginseng Radix et Rhizoma, Astragali Radix, Polygalae Radix, Poria, Rehmanniae Radix* |
| Researches on traditional Chinese medicine formulas for treating senile cognitive impairment based on traditional Chinese medicine inheritance auxiliary systems (Xia, 2015) | *Acori Tatarinowii Rhizoma, Chuanxiong Rhizoma, Polygalae Radix, Salviae Miltiorrhizae Radix et Rhizoma, Astragali Radix, Rehmanniae Radix, Poria, Angelicae Sinensis Radix, Curcumae Rhizoma, Corni Fructus* |
| Studies on the rule of compound traditional Chinese medicine for treating Alzheimer's disease (Zhou et al., 2005) | *Acori Tatarinowii Rhizoma, Chuanxiong Rhizoma, Polygalae Radix, Salviae Miltiorrhizae Radix et Rhizoma, Rehmanniae Radix, Angelicae Sinensis Radix, Lycii Fructus, Poria, Astragali Radix, Polygoni Multiflori Radix* |
| Analysis of medication rules for the treatment of Alzheimer’s disease (Deng et al., 2007) | *Acori Tatarinowii Rhizoma, Polygoni Multiflori Radix, Polygalae Radix, Rehmanniae Radix, Salviae Miltiorrhizae Radix et Rhizoma, Chuanxiong Rhizoma, Astragali Radix, Poria, Lycii Fructus, Angelicae Sinensis Radix* |
| Studies on medication rules for the treatment of Alzheimer’s disease in Ming and Qing Dynasties(Wang, 2010) | *Ginseng Radix et Rhizoma, Polygalae Radix, Acori Tatarinowii Rhizoma, Poria, Glycyrrhizae Radix et Rhizoma, Poria cum Radix Pini, Ophiopogonis Radix, Angelicae Sinensis Radix, Cinnabar, Rehmanniae Radix* |
| Analysis of randomly controlled literature therapeutics and medication rules of traditional Chinese medicine for Alzheimer’s disease (Han et al., 2014) | *Acori Tatarinowii Rhizoma, Chuanxiong Rhizoma, Salviae Miltiorrhizae Radix et Rhizoma, Polygalae Radix, Polygoni Multiflori Radix, Corni Fructus, Poria, Lycii Fructus, Rehmanniae Radix, Alpiniae Oxyphyllae Fructus* |
| Analysis of medication rules of compound traditional Chinese medicine in treating Alzheimer’s disease (Hu et al., 2012) | *Acori Tatarinowii Rhizoma, Chuanxiong Rhizoma, Astragali Radix, Polygoni Multiflori Radix, Ginseng Radix et Rhizoma, Salviae Miltiorrhizae Radix et Rhizoma, Polygalae Radix, Rehmanniae Radix, Poria, Lycii Fructus* |
| Studies on the medication rules of the literature on the treatment of Alzheimer’s disease with traditional Chinese medicine (Yan, 2007) | *Acori Tatarinowii Rhizoma, Rehmanniae Radix, Salviae Miltiorrhizae Radix et Rhizoma, Polygalae Radix, Astragali Radix, Corni Fructus, Polygoni Multiflori Radix, Lycii Fructus, Chuanxiong Rhizoma, Angelicae Sinensis Radix* |
| Exploring compatibility of prescriptions of traditional Chinese medicine for the treatment of Alzheimer's Disease (Wei et al., 2015) | *Acori Tatarinowii Rhizoma, Polygalae Radix, Rehmanniae Radix, Polygoni Multiflori Radix, Angelicae Sinensis Radix, Chuanxiong Rhizoma, Astragali Radix, Corni Fructus, Poria, Alpiniae Oxyphyllae Fructus* |

**Table S2. The appearance frequency of TCMs in the literature against dementia.** TCMs listed in Table S1 were further sorted according to their occurrences, 24 different TCM herbal medicines were obtained, 19 of them appear more than twice. Since *Cinnabar* mainly composed of mercury sulfide, *Ginkgo biloba* was used instead.

| **Traditional Chinese medicines** | **Frequency** | **Traditional Chinese medicines** | **Frequency** | **Traditional Chinese medicines** | **Frequency** |
| --- | --- | --- | --- | --- | --- |
| *Polygalae Radix*(Wang et al., 2019;Kuboyama et al., 2017) | 20 | *Lycii Fructus*(Hu et al., 2018;Zhou et al., 2018) | 10 | *Curcumae Rhizoma*(da Costa et al., 2019;Ringman et al., 2012) | 2 |
| *Acori Tatarinowii Rhizoma*(Yang et al., 2017;Geng et al., 2010) | 20 | *Polygoni Multiflori Radix*(Ling and Xu, 2016;Jiao et al., 2018) | 9 | *Alpiniae Oxyphyllae Fructus*(Qi et al., 2019;He et al., 2019) | 2 |
| *Poria* (May et al., 2016;Fu et al., 2016) | 19 | *Ginseng Radix et Rhizoma*(Kim et al., 2018;Rajabian et al., 2019) | 8 | *Cinnabar* | 2 |
| *Rehmanniae Radix* (Lee et al., 2011;Liu et al., 2018) | 19 | *Glycyrrhizae Radix et Rhizoma* (Link et al., 2015;Gu et al., 2018) | 8 | *Pinelliae Rhizoma* | 1 |
| *Angelicae Sinensis Radix*(Du et al., 2020;Duan et al., 2016) | 18 | *Corni Fructus*(Zhao et al., 2010;Yang et al., 2020) | 7 | *Ginkgo biloba*(Savaskan et al., 2018;von Gunten et al., 2016) | 1 |
| *Chuanxiong Rhizoma*(Fu et al., 2016;Dai et al., 2016) | 16 | *Atractylodis Macrocephalae Rhizoma*(Zhu et al., 2018) | 3 | *Dioscoreae Rhizoma* | 1 |
| *Astragali Radix*(Huang et al., 2017;Zhang et al., 2018) | 13 | *Poria cum Radix Pini*(Hou et al., 2014) | 3 | *Os Draconis* | 1 |
| *Salviae Miltiorrhizae Radix et Rhizoma*(Zhang et al., 2016b;Chong et al., 2019) | 13 | *Ophiopogonis Radix*(Qu et al., 2011;Zhang et al., 2016a) | 3 | *Arisaematis Rhizoma* | 1 |

**Table S3. Active chemicals of selected 19 TCMs.** 10 top-reported active chemicals from these 19 TCMs through literature investigation were selected, 116 of these 190 chemicals were commercially available, and 40 of the 116 chemicals had been reported to show effects on protein aggregation, therefore, further studies were performed with the rest 76 chemicals.

| **Herb name** | **Active chemicals** |
| --- | --- |
| *Polygalae Radix* | Alizarin^a^, Isofraxidin^a^, Rubiadin^a^, 1-Hydroxyanthraquinone^a^, 2-Methylanthraquinone^a^, Luteolin (Ali and Siddique, 2019), Tenuifolin (Deng et al., 2020), Tetrahydrocolumbamine^c^, Onjisaponin B^c^, Polygalacin D^c^ |
| *Acori Tatarinowii Rhizoma* | Apigenin^a^, Astragaline^a^, Isopimpinellin^a^, Methyleugenol^a^, Rhoifolin^a^, Thymol^a^, α-Asarone^a^, 2,4,5-Trimethoxybenzoic acid^a^, 2,6-Dimethoxy-p-Quinone^a^, Cinnamic acid^b^ (Tsunoda et al., 2018) |
| *Poria* | Uridine^a^, Paeoniflorin^a^, Tumulosic acid^c^, 2,5-Methoxyporicoic acid A^c^, 16-Deoxyporicoic acid B^c^, 3-O-Acetyl-16α-hydroxytrametenolic acid^c^, Pachymic acid^c^, Dehydrotrametenolic acid^c^, Dehydropachymic acid^c^, Poricoic acid A^c^ |
| *Rehmanniae Radix* | Aucubin^a^, Echinacoside^a^, Forsythoside B^a^, Geniposide^a^, Loganic acid^a^, Rehmannioside D^a^, Verbascoside^b^ (Korshavn et al., 2015), Catalpol^b^ (Dinda et al., 2019), Ajugoside^c^, 8-Epiloganic acid^c^ |
| *Angelicae Sinensis Radix* | Isoeugenol^a^, Osthole^a^, Scopoletin^a^, 2-Acetyl-4-Methylphenol^a^, 4-Hydroxyindole^a^, Ferulic acid^b^(Sgarbossa et al., 2013), Bergapten^c^, Isoacroraene^c^, Chamigrene^c^, Z-ligustilide dimer E-232^c^ |
| *Chuanxiong Rhizoma* | Ligustilide^a^, Sinapic acid^a^, 2-Methoxy-4-propylphenol^a^, 2-Methylbenzoxazole^a^, Ligustrazine^b^ (Wu et al., 2018), Ferulic acid^b^ (Askar et al., 2019), Chuanxiongoside A^c^, Chuanxiongoside B^c^, L-maackiain^c^, Senkyunolide E^c^ |
| *Astragali Radix* | Calycosin 7-O-glucoside^a^, Engenol^a^, Epiberberine^a^, Ononin^a^, Oroxylin A^a^, Baicalein^b^(Hung et al., 2016), Kaempferol^b^ (Hanaki et al., 2016), Isorhamnetin^b^ (Hanaki et al., 2016), Kumatakenin^c^, Astragaloside^c^ |
| *Salviae miltiorrhizae Radix et Rhizoma* | Cyanidol,^a^ Dihydrotanshinone^a^, Isoimperatorin^a^, 3-(3,4-dihydroxyphenyl)-DL-lactate^a^, 3,4-Dihydroxybenzaldehyde^a^, Caffeic acid^b^(Cheng et al., 2013), Protocatechuic acid^b^ (Hornedo-Ortega et al., 2016), Salvianolic acid^b^ (Wu et al., 2019), Tanshinone^b^ (Ji et al., 2016) , Aethiopinone^c^ |
| *Lycii Fructus* | Coumalic acid^a^, Glycitein^a^, Scopolin^a^, 6,7-Dihydroxycoumarin^a^, Hypaconitine^c^, Daucosterol^c^, Campesteryl ferulate^c^, Darutoside^c^, Isoscopoletin^c^, Isoferulic acid^c^ |
| *Polygoni Multiflori Radix* | Chrysophanol^a^, Schizandrin B^a^, 4-Hydroxy benzaldehyde^a^, 2,3,5,4'-tetrahydroxyl diphenylethylene-2-o-glucoside^a^, Emodin^b^ (Wang et al., 2020), Parietin^b^ (Cornejo et al., 2016), Lecithin^b^ (Aoyagi et al., 2015), (±)-Catechin^b^ (Xie et al., 2014), (-)-Epicatechin^b^(Rho et al., 2019), Rhein^b^(Ho et al., 2015) |
| *Ginseng Radix et Rhizoma* | Dauricine^a^, Paeonol^a^, Ginsenoside Rb1^b^(Ardah et al., 2015), Ginsenoside Rb2^b^, Ginsenoside Rb3^b^, Ginsenoside Rc^b^, Ginsenoside Rg1^b^(Zheng et al., 2019), Ginsenoside Rg3^b^, Ginsenoside Rh2^b^(Rajabian et al., 2019), Ginseng polysaccharide^c^ |
| *Glycyrrhizae Radix et Rhizoma* | Puerarin^a^, Calcium folinate^a^, Isoliquiritigenin^b^ (Link et al., 2015), Liquiritin^b^ (Jia et al., 2016), Liquiritigenin^b^ (Link et al., 2015), Morusin^c^, Narcissoside^c^, Castanin^c^, Semilicoisoflavone B^c^, Liconeolignan^c^ |
| *Corni Fructus* | Benzyl cinnamate^a^, Ethylvanillin^a^, Loganin^a^, Naringenin^a^, Swertiamarine^a^, 3,5-Dihydroxybenzoic acid^a^, 3-O-Methylgallic acid^a^, Oleanolic acid^b^ (Fujihara et al., 2017), Gallic acid^b^ (Yu et al., 2019), Cornuside^c^ |
| *Atractylodis Macrocephalae Rhizoma* | Icariin^a^, Evodiamine^a^, α-tractlone^c^, (+)-α-curcumene^c^, Selina-4(15),7(11)-dien-8-one^c^, 3β-acetoxyatractylone^c^, 8β-ethoxyatractylenolide-Ⅱ^c^, Atractylenolide-Ⅰ^c^, Atractylenolide-Ⅱ^c^, Atractylenolide-Ⅲ^c^ |
| *Poria cum Radix Pini* | Gastrodin^a^, Vanillicalcohol^a^, Vanillin^b^ (Iannuzzi et al., 2017), Polyporenic acid^c^, Eburicoic acid^c^, Trametenolic acid^c^, Dehydrotumulosic acid^c^, Dehydroeburicoic acid^c^, O-Acetylpachymic acid^c^, 7,9(11)-dehydropachymic acid^c^ |
| *Ophiopogonis Radix* | Isovanillin^a^, Tectochrysin^a^, Vanillic acid^b^ (Amin et al., 2017), Jasmolone^c^, Ophiopogonanone^c^, β-sitosteryl-β-D-glucopyranoside^c^, Desmethylisoophiopogonone^c^, Methylophiopogonanone^c^, Ophiopogonin B^c^, Ophiopogonin D^c^ |
| *Curcumae Rhizoma.* | Schizandrol A^a^, Tetrandrine^a^, Quercetin^b^ (Khan et al., 2019), Tuliposide A^c^, Tuliposide B^c^, Tulipzlin^c^, Curcumanolide A^c^, Alnusone^c^, Sitogluside^c^, Furanogermenone^c^ |
| *Alpiniae Oxyphyllae Fructus* | Polydatin^a^, Galangin^a^, Yakuchinone A^c^, Bullatantriol^c^, Dihydrogingerenone B^c^, Teucrenone^c^, Isalpinin^c^, Rhamnocitrin^c^, Stigmasterol^c^, Oxyphyllacinol^c^ |
| *Ginkgo biloba* | Anethol^a^, Diosmetin^a^, Phthalide^a^, Rutin^a^, Sesamin^a^, Soy Isoflavones^a^, Ginkgolide B^b^ (Huang et al., 2017), Ginkgolide J^b^, Ginkgolide C^b^, Ginkgolide A^b^ (Guo et al., 2010) |

^a^ Chemicals commercially purchased

^b^ Chemicals have been reported to affect protein aggregation

^c^ Chemicals not commercially available**Table S4. The plant source, chemical name, commercial source, structure, cytotoxicity *per se*, and effects** **on Aβ42 cytotoxicity of the 76 chemicals screened.** 26 of these chemicals exhibited cytotoxicity below 10% at the concentration of 20 μM. 24 chemicals showed significantly enhanced toxicity or no obvious effects when co-incubated with Aβ42, and tetrahydroxystilbene-2-O-β-D-glucoside (TSG) and sinapic acid (SA) significantly attenuated the cytotoxicity of Aβ42.

| **Herb name** | **Chemical** | **Commercialization source** | **Structure** | **Cytotoxicity (20 μM)** | **Inhibit Aβ42 cytotoxicity(20 μM)** |
| --- | --- | --- | --- | --- | --- |
| *Polygalae Radix* | Alizarin | Aladdin-Reagents (Shanghai, China) | 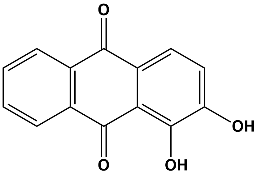 | 19.4 ± 2.6% | / |
|  | Isofraxidin | Yuanye Biotech. (Shanghai, China) | 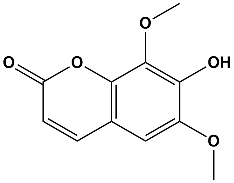 | 13.3 ± 2.6% | / |
|  | Rubiadin | Yuanye Biotech. (Shanghai, China) | 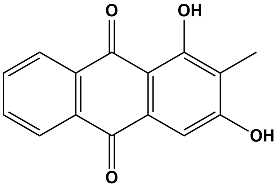 | 10.6 ± 3.2% | / |
|  | 1-Hydroxyanthraquinone | Aladdin-Reagents (Shanghai, China) | 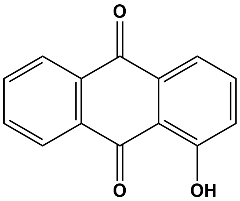 | 1.9 ± 5.4% | -0.8 ± 2.3% |
|  | 2-Methylanthraquinone | Aladdin-Reagents (Shanghai, China) | 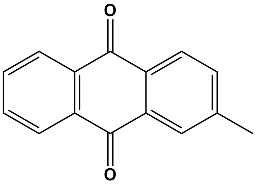 | 15.2 ± 2.4% | / |
| *Acori Tatarinowii Rhizoma* | Apigenin | Aladdin-Reagents (Shanghai, China) | 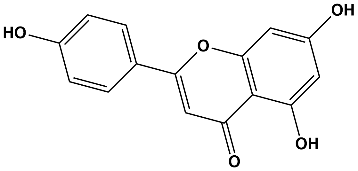 | 28.0 ± 2.1% | / |
|  | Astragaline | Yuanye Biotech. (Shanghai, China) | 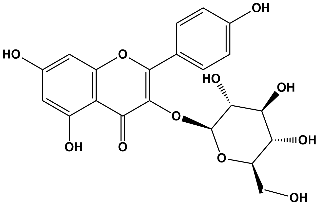 | 5.6 ± 2.0% | +0.3 ± 2.9% |
|  | Isopimpinellin | Yuanye Biotech. (Shanghai, China) | 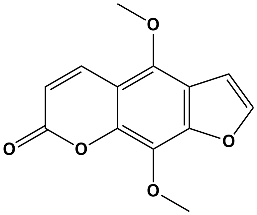 | 21.1 ± 2.5% | / |
|  | Methyleugenol | Aladdin-Reagents (Shanghai, China) | 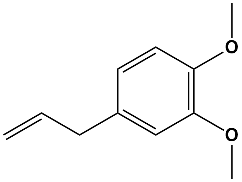 | 5.9 ± 3.1% | -5.7 ± 2.8% |
|  | Rhoifolin | Yuanye Biotech. (Shanghai, China) | 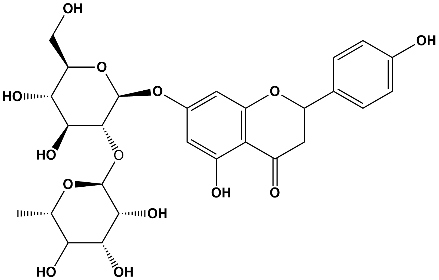 | 14.3 ± 3.1% | / |
|  | Thymol | Aladdin-Reagents (Shanghai, China) | 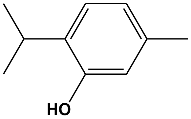 | 6.8 ± 1.9% | -4.7 ± 2.4% |
|  | α-Asarone | Yuanye Biotech. (Shanghai, China) | 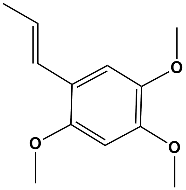 | 17.1 ± 2.6% | / |
|  | 2,4,5-Trimethoxybenzoic acid | Aladdin-Reagents (Shanghai, China) | 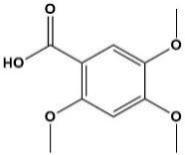 | 7.0 ± 4.2% | -2.2 ± 4.3% |
|  | 2,6-Dimethoxy-p-Quinone | Yuanye Biotech. (Shanghai, China) | 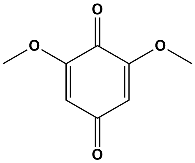 | 42.7 ± 3.3% | / |
| *Poria* | Uridine | Aladdin-Reagents (Shanghai, China) | 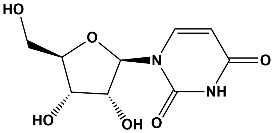 | -3.4 ± 2.5% | -4.3 ± 2.8% |
|  | Paeoniflorin | Aladdin-Reagents (Shanghai, China) | 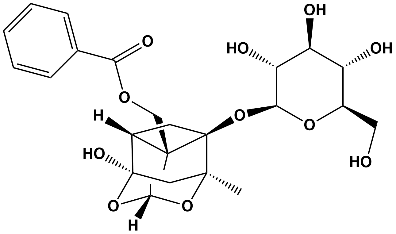 | 17.8 ± 5.8% | / |
| *Rehmanniae Radix* | Aucubin | Yuanye Biotech. (Shanghai, China) | 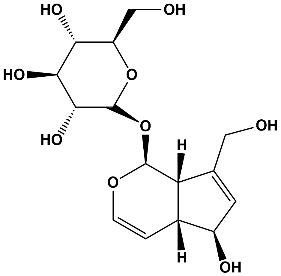 | 9.2 ± 2.7% | -1.9 ± 2.5% |
|  | Echinacoside | Yuanye Biotech. (Shanghai, China) | 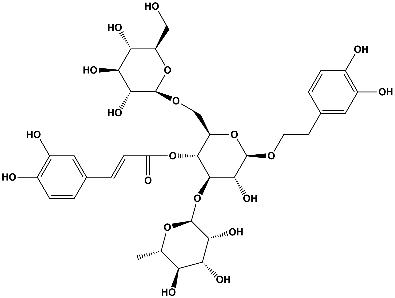 | 22.5 ± 3.0% | / |
|  | Forsythoside B | Yuanye Biotech. (Shanghai, China) | 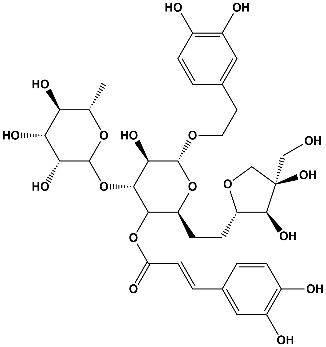 | 22.9 ± 4.9% | / |
|  | Geniposide | Aladdin-Reagents (Shanghai, China) | 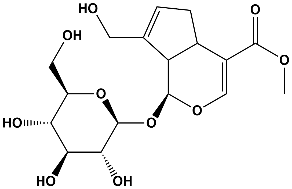 | 16.7 ± 2.5% | / |
|  | Loganic acid | Yuanye Biotech. (Shanghai, China) | 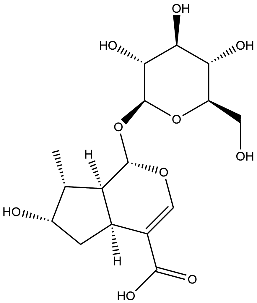 | 13.5 ± 3.1% | / |
|  | Rehmannioside D | Yuanye Biotech. (Shanghai, China) | 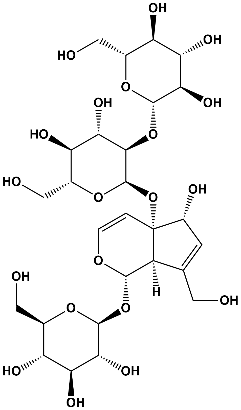 | 7.8 ± 2.9% | -2.1 ± 2.2% |
| *Angelicae Sinensis Radix* | Isoeugenol | Aladdin-Reagents (Shanghai, China) | 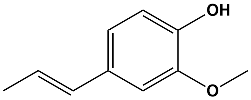 | 6.1 ± 1.9% | 0.0 ± 4.0% |
|  | Osthole | Yuanye Biotech. (Shanghai, China) | 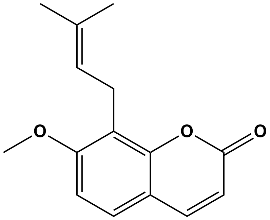 | 5.9 ± 3.7% | -1.9 ± 5.1% |
|  | Scopoletin | Yuanye Biotech. (Shanghai, China) | 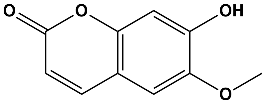 | 11.9 ± 1.3% | / |
|  | 2-Acetyl-4-Methylphenol | Aladdin-Reagents (Shanghai, China) | 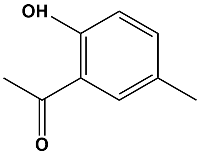 | 6.5 ± 1.9% | -2.7 ± 1.3% |
|  | 4-Hydroxyindole | Yuanye Biotech. (Shanghai, China) | 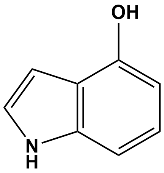 | 1.2 ± 3.0% | +2.6 ± 7.3% |
| *Chuanxiong Rhizoma* | Ligustilide | Aladdin-Reagents (Shanghai, China) | 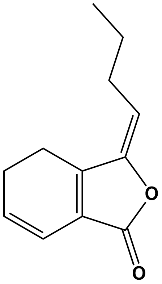 | 2.1 ± 3.2% | -2.1 ± 2.6% |
|  | Sinapic acid | Aladdin-Reagents (Shanghai, China) | 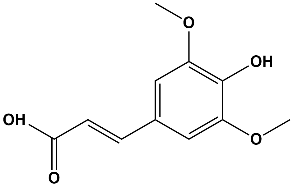 | -2.0 ± 4.9% | +8.8 ± 3.6% |
|  | 2-Methoxy-4-propylphenol | Aladdin-Reagents (Shanghai, China) | 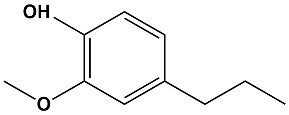 | 6.9 ± 3.3% | -8.7 ± 0.7% |
|  | 2-Methylbenzoxazole | Aladdin-Reagents (Shanghai, China) | 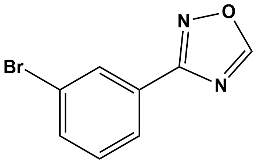 | 9.5 ± 2.3% | -4.4 ± 4.6% |
| *Astragali Radix* | Calycosin 7-O-glucoside | Yuanye Biotech. (Shanghai, China) | 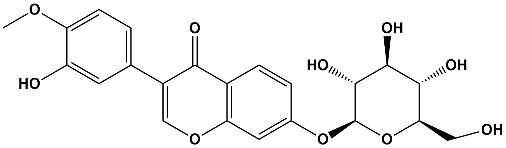 | 12.0 ± 1.7% | / |
|  | Engenol | Aladdin-Reagents (Shanghai, China) | 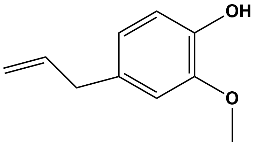 | 4.7 ± 3.0% | -1.1 ± 1.2% |
|  | Epiberberine | Yuanye Biotech. (Shanghai, China) | 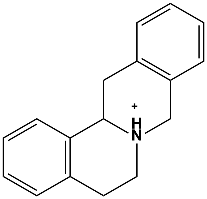 | 23.2 ± 4.3% | / |
|  | Ononin | Aladdin-Reagents (Shanghai, China) | 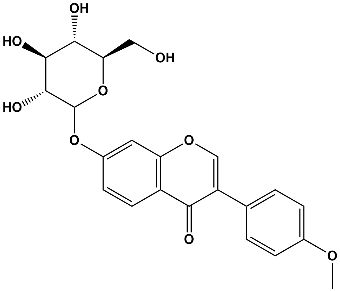 | 12.9 ± 3.6% | / |
|  | Oroxylin A | Yuanye Biotech. (Shanghai, China) | 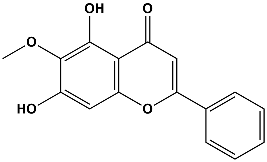 | 12.7 ± 1.7% | / |
| *Salviae Miltiorrhizae Radix et Rhizoma* | Cyanidol | Yuanye Biotech. (Shanghai, China) | 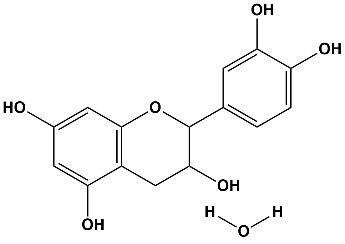 | 20.2 ± 3.4% | / |
|  | Dihydrotanshinone | Yuanye Biotech. (Shanghai, China) | 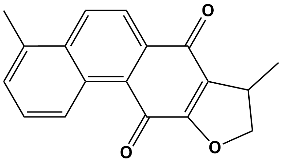 | 12.2 ± 4.5% | / |
|  | Isoimperatorin | Yuanye Biotech. (Shanghai, China) | 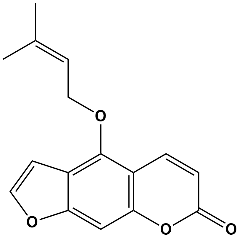 | 19.4 ± 1.9% | / |
|  | 3-(3,4-dihydroxyphenyl)-DL-lactate | Aladdin-Reagents (Shanghai, China) | 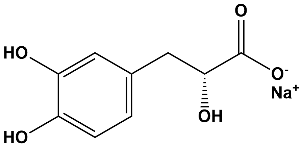 | 23.0 ± 4.6% | / |
|  | 3,4-Dihydroxybenzaldehyde | Aladdin-Reagents (Shanghai, China) | 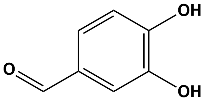 | 12.2 ± 4.5% | / |
| *Lycii Fructus* | Coumalic acid | Aladdin-Reagents (Shanghai, China) | 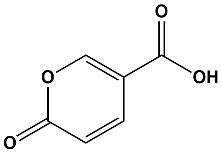 | 0.1 ± 2.5% | +2.1 ± 4.5% |
|  | Glycitein | Yuanye Biotech. (Shanghai, China) | 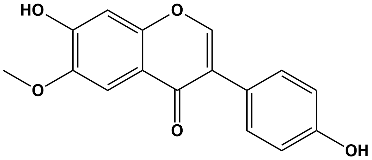 | 17.5 ± 1.2% | / |
|  | Scopolin | Yuanye Biotech. (Shanghai, China) | 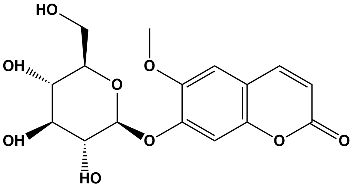 | 11.9 ± 1.3% | -6.5 ± 1.9% |
|  | 6,7-Dihydroxycoumarin | Aladdin-Reagents (Shanghai, China) | 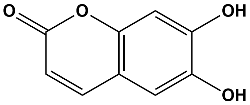 | 1.7 ± 7.1% | -14.7 ± 3.7% |
| *Polygoni Multiflori Radix* | Chrysophanol | Yuanye Biotech. (Shanghai, China) | 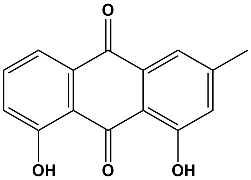 | 11.6 ± 2.9% | / |
|  | Schizandrin B | Yuanye Biotech. (Shanghai, China) | 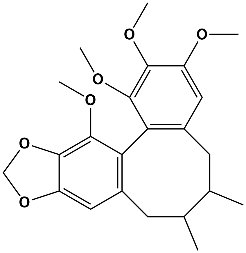 | 15.2 ± 2.7% | / |
|  | 4-Hydroxy benzaldehyde | Aladdin-Reagents (Shanghai, China) | 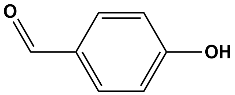 | 14.6 ± 2.6% | / |
|  | 2,3,5,4'-tetrahydroxyl diphenylethylene-2-o-glucoside | Yuanye Biotech. (Shanghai, China) | 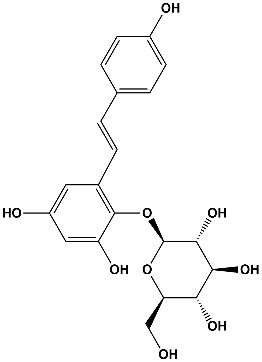 | 2.9 ± 3.3% | +4.1 ± 2.4% |
| *Ginseng Radix et Rhizoma* | Dauricine | Yuanye Biotech. (Shanghai, China) | 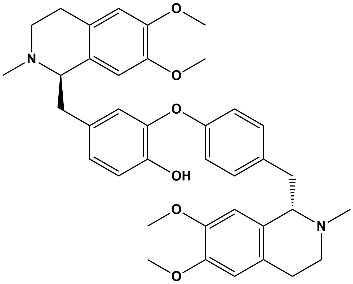 | 22.9 ± 2.0% | / |
|  | Paeonol | Aladdin-Reagents (Shanghai, China) | 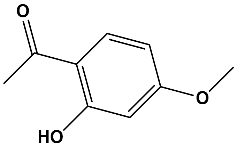 | 6.3 ± 1.3% | -2.6 ± 2.3% |
| *Glycyrrhizae Radix et Rhizoma* | Puerarin | Yuanye Biotech. (Shanghai, China) | 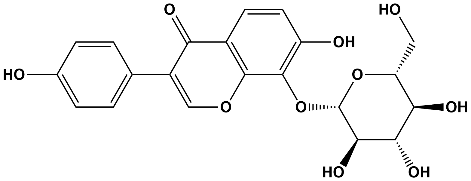 | 13.1 ± 4.2% | / |
|  | Calcium folinate | Aladdin-Reagents (Shanghai, China) | 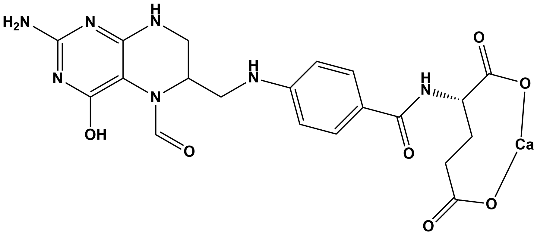 | 13.4 ± 2.1% | / |
| *Corni Fructus* | Benzyl cinnamate | Aladdin-Reagents (Shanghai, China) | 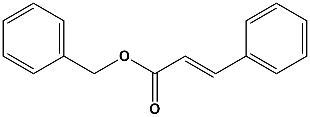 | 12.3 ± 4.8% | / |
|  | Ethylvanillin | Aladdin-Reagents (Shanghai, China) | 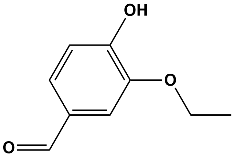 | 18.7 ± 3.1% | / |
|  | Loganin | Yuanye Biotech. (Shanghai, China) | 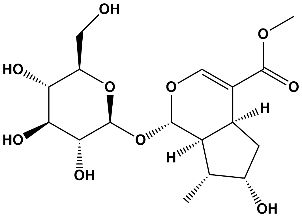 | 2.4 ± 1.9% | -2.8 ± 3.1% |
|  | Naringenin | Aladdin-Reagents (Shanghai, China) | 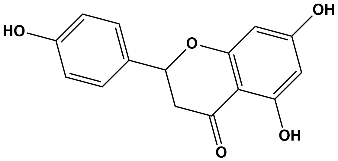 | 13.7 ± 2.4% | / |
|  | Swertiamarine | Aladdin-Reagents (Shanghai, China) | 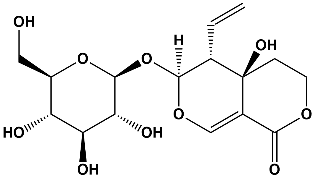 | 6.2 ± 1.0% | -9.3 ± 1.8% |
|  | 3,5-Dihydroxybenzoic acid | Aladdin-Reagents (Shanghai, China) | 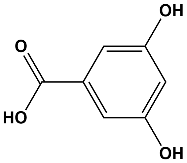 | 12.6 ± 3.2% | / |
|  | 3-O-Methylgallic acid | Yuanye Biotech. (Shanghai, China) | 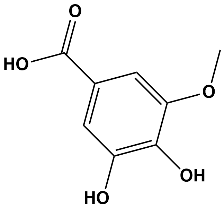 | 24.9 ± 3.1% | / |
| *Atractylodis macrocephalae Rhizoma* | Icariin | Aladdin-Reagents (Shanghai, China) | 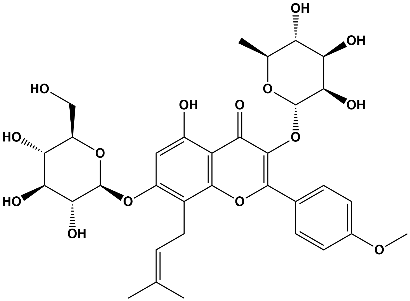 | 27.4 ± 1.1% | / |
|  | Evodiamine | Aladdin-Reagents (Shanghai, China) | 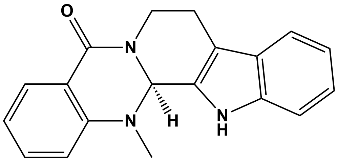 | 36.2 ± 2.7% | / |
| *Poria cum Radix Pini* | Gastrodin | Yuanye Biotech. (Shanghai, China) | 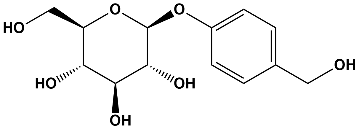 | 18.8 ± 1.8% | / |
|  | Vanillicalcohol | Aladdin-Reagents (Shanghai, China) | 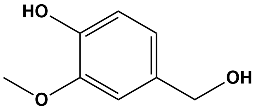 | 13.7 ± 4.6% | / |
| *Ophiopogonis Radix* | Isovanillin | Aladdin-Reagents (Shanghai, China) | 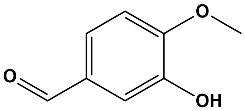 | 16.0 ± 2.5% | / |
|  | Tectochrysin | Yuanye Biotech. (Shanghai, China) | 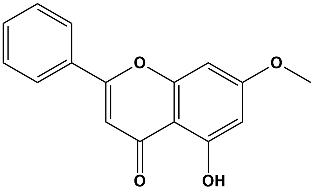 | 17.5 ± 2.5% | / |
| *Curcumae Rhizoma.* | Schizandrol A | Yuanye Biotech. (Shanghai, China) | 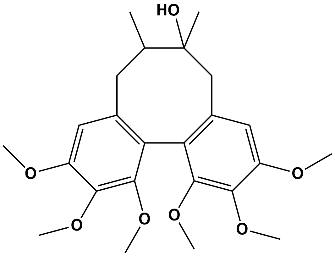 | 18.0 ± 2.1% | / |
|  | Tetrandrine | Aladdin-Reagents (Shanghai, China) | 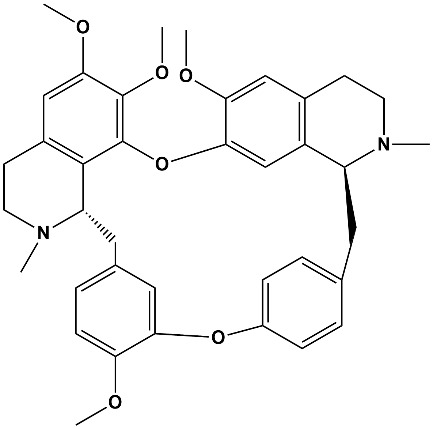 | 30.1 ± 1.6% | / |
| *Alpiniae Oxyphyllae Fructus* | Polydatin | Aladdin-Reagents (Shanghai, China) | 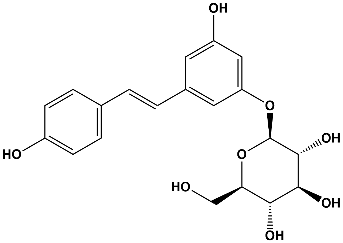 | 25.1 ± 1.9% | / |
|  | Galangin | Aladdin-Reagents (Shanghai, China) | 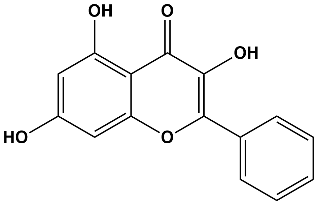 | 23.8 ± 3.8% | / |
| *Ginkgo biloba* | Anethol | Yuanye Biotech. (Shanghai, China) | 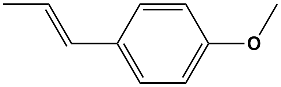 | 15.8 ± 1.6% | / |
|  | Diosmetin | Yuanye Biotech. (Shanghai, China) | 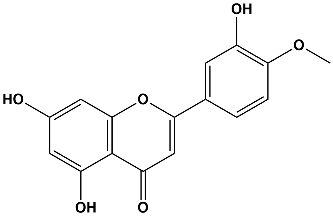 | 28.7 ± 2.0% | / |
|  | Phthalide | Aladdin-Reagents (Shanghai, China) | 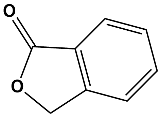 | 3.3 ± 2.3% | -7.7 ± 3.3% |
|  | Rutin | Yuanye Biotech. (Shanghai, China) | 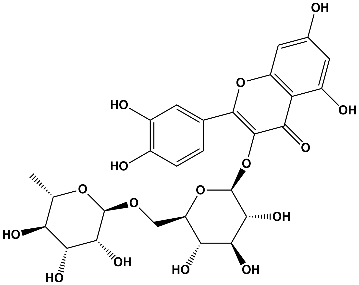 | 18.4 ± 2.1% | / |
|  | Sesamin | Aladdin-Reagents (Shanghai, China) | 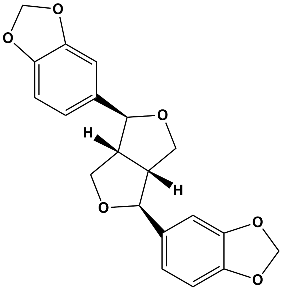 | 12.8 ± 2.0% | / |
|  | Soy Isoflavones | Yuanye Biotech. (Shanghai, China) | 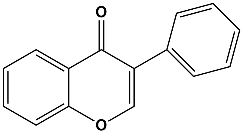 | 9.6 ± 3.2% | -7.3 ± 2.2% |

**References**

Ali, F., and Siddique, Y.H. (2019). Bioavailability and Pharmaco-therapeutic Potential of Luteolin in Overcoming Alzheimer's Disease. *CNS Neurol Disord Drug Targets* 18(5)**,** 352-365. doi: 10.2174/1871527318666190319141835.

Amin, F.U., Shah, S.A., and Kim, M.O. (2017). Vanillic acid attenuates Abeta1-42-induced oxidative stress and cognitive impairment in mice. *Sci Rep* 7**,** 40753. doi: 10.1038/srep40753.

Aoyagi, S., Shimanouchi, T., Kawashima, T., and Iwai, H. (2015). ToF-SIMS observation for evaluating the interaction between amyloid beta and lipid membranes. *Anal Bioanal Chem* 407(10)**,** 2859-2863. doi: 10.1007/s00216-015-8527-3.

Ardah, M.T., Paleologou, K.E., Lv, G., Menon, S.A., Abul Khair, S.B., Lu, J.H., et al. (2015). Ginsenoside Rb1 inhibits fibrillation and toxicity of alpha-synuclein and disaggregates preformed fibrils. *Neurobiol Dis* 74**,** 89-101. doi: 10.1016/j.nbd.2014.11.007.

Askar, M.H., Hussein, A.M., Al-Basiony, S.F., Meseha, R.K., Metias, E.F., Salama, M.M., et al. (2019). Effects of Exercise and Ferulic Acid on Alpha Synuclein and Neuroprotective Heat Shock Protein 70 in An Experimental Model of Parkinsonism Disease. *CNS Neurol Disord Drug Targets* 18(2)**,** 156-169. doi: 10.2174/1871527317666180816095707.

Cheng, B., Gong, H., Xiao, H., Petersen, R.B., Zheng, L., and Huang, K. (2013). Inhibiting toxic aggregation of amyloidogenic proteins: a therapeutic strategy for protein misfolding diseases. *Biochim Biophys Acta* 1830(10)**,** 4860-4871. doi: 10.1016/j.bbagen.2013.06.029.

Chong, C.M., Su, H., Lu, J.J., and Wang, Y. (2019). The effects of bioactive components from the rhizome of Salvia miltiorrhiza (Danshen) on the characteristics of Alzheimer's disease. *Chin Med* 14**,** 19. doi: 10.1186/s13020-019-0242-0.

Cornejo, A., Salgado, F., Caballero, J., Vargas, R., Simirgiotis, M., and Areche, C. (2016). Secondary Metabolites in Ramalina terebrata Detected by UHPLC/ESI/MS/MS and Identification of Parietin as Tau Protein Inhibitor. *Int J Mol Sci* 17(8). doi: 10.3390/ijms17081303.

da Costa, I.M., Freire, M.A.M., de Paiva Cavalcanti, J.R.L., de Araujo, D.P., Norrara, B., Moreira Rosa, I.M.M., et al. (2019). Supplementation with Curcuma longa Reverses Neurotoxic and Behavioral Damage in Models of Alzheimer's Disease: A Systematic Review. *Curr Neuropharmacol* 17(5)**,** 406-421. doi: 10.2174/0929867325666180117112610.

Dai, Y., Ma, T., Ren, X., Wei, J., Fu, W., Ma, Y., et al. (2016). Tongluo Xingnao Effervescent Tablet preserves mitochondrial energy metabolism and attenuates cognition deficits in APPswe/PS1De9 mice. *Neurosci Lett* 630**,** 101-108. doi: 10.1016/j.neulet.2016.07.044.

Deng, J,; Hao, E.; Guo, H.; Liu, J., Analysis of drug law of compound prescription in treating Alzheimer disease. Shandong Journal of Traditional Chinese Medicine 2007, 363-365.

Deng, X., Zhao, S., Liu, X., Han, L., Wang, R., Hao, H., et al. (2020). Polygala tenuifolia: a source for anti-Alzheimer's disease drugs. *Pharm Biol* 58(1)**,** 410-416. doi: 10.1080/13880209.2020.1758732.

Dinda, B., Dinda, M., Kulsi, G., Chakraborty, A., and Dinda, S. (2019). Therapeutic potentials of plant iridoids in Alzheimer's and Parkinson's diseases: A review. *Eur J Med Chem* 169**,** 185-199. doi: 10.1016/j.ejmech.2019.03.009.

Du, Q., Zhu, X., and Si, J. (2020). Angelica polysaccharide ameliorates memory impairment in Alzheimer's disease rat through activating BDNF/TrkB/CREB pathway. *Exp Biol Med (Maywood)* 245(1)**,** 1-10. doi: 10.1177/1535370219894558.

Duan, M.H., Wang, L.N., Jiang, Y.H., Pei, Y.Y., Guan, D.D., and Qiu, Z.D. (2016). Angelicae Sinensis Radix reduced Abeta-induced memory impairment in rats. *J Drug Target* 24(4)**,** 340-347. doi: 10.3109/1061186X.2015.1077848.

Fan, T.; Wang, H.; Ma, F.; Bai, Y.; Yang, Y.; Chen, Y., Research on the rule mining of Chinese medicine compound patent treatment Alzheimer's disease based on sooPAT search engine. Traditional Chinese Medicine Journal 2019, 18, 40-43+53.

Fu, X., Wang, Q., Wang, Z., Kuang, H., and Jiang, P. (2016). Danggui-Shaoyao-San: New Hope for Alzheimer's Disease. *Aging Dis* 7(4)**,** 502-513. doi: 10.14336/AD.2015.1220.

Fujihara, K., Koike, S., Ogasawara, Y., Takahashi, K., Koyama, K., and Kinoshita, K. (2017). Inhibition of amyloid beta aggregation and protective effect on SH-SY5Y cells by triterpenoid saponins from the cactus Polaskia chichipe. *Bioorg Med Chem* 25(13)**,** 3377-3383. doi: 10.1016/j.bmc.2017.04.023.

Geng, Y., Li, C., Liu, J., Xing, G., Zhou, L., Dong, M., et al. (2010). Beta-asarone improves cognitive function by suppressing neuronal apoptosis in the beta-amyloid hippocampus injection rats. *Biol Pharm Bull* 33(5)**,** 836-843. doi: 10.1248/bpb.33.836.

Gu, M.Y., Chun, Y.S., Zhao, D., Ryu, S.Y., and Yang, H.O. (2018). Glycyrrhizae Radix et Rhizoma and Semilicoisoflavone B Reduce Abeta Secretion by Increasing PPARgamma Expression and Inhibiting STAT3 Phosphorylation to Inhibit BACE1 Expression. *Mol Nutr Food Res* 62(6)**,** e1700633. doi: 10.1002/mnfr.201700633.

Guo, J.P., Yu, S., and McGeer, P.L. (2010). Simple in vitro assays to identify amyloid-beta aggregation blockers for Alzheimer's disease therapy. *J Alzheimers Dis* 19(4)**,** 1359-1370. doi: 10.3233/JAD-2010-1331.

Hanaki, M., Murakami, K., Akagi, K., and Irie, K. (2016). Structural insights into mechanisms for inhibiting amyloid beta42 aggregation by non-catechol-type flavonoids. *Bioorg Med Chem* 24(2)**,** 304-313. doi: 10.1016/j.bmc.2015.12.021.

Han, D.; Yang, X.; Shi, J.; Tian, J., Analysis on Therapies and Medications in Randomized Controlled Trials of TCM for Dementia. *Journal of Traditional Chinese Medicine* 2014, ***55***, 1051-1054.

He, B., Xu, F., Yan, T., Xiao, F., Wu, B., Wang, Y., et al. (2019). Tectochrysin from Alpinia Oxyphylla Miq. alleviates Abeta1-42 induced learning and memory impairments in mice. *Eur J Pharmacol* 842**,** 365-372. doi: 10.1016/j.ejphar.2018.11.002.

Ho, S.L., Poon, C.Y., Lin, C., Yan, T., Kwong, D.W., Yung, K.K., et al. (2015). Inhibition of beta-amyloid Aggregation by Albiflorin, Aloeemodin And Neohesperidin And Their Neuroprotective Effect On Primary Hippocampal Cells Against beta-amyloid Induced Toxicity. *Curr Alzheimer Res* 12(5)**,** 424-433. doi: 10.2174/1567205012666150504144919.

Hornedo-Ortega, R., Alvarez-Fernandez, M.A., Cerezo, A.B., Richard, T., Troncoso, A.M.A., and Garcia-Parrilla, M.A.C. (2016). Protocatechuic Acid: Inhibition of Fibril Formation, Destabilization of Preformed Fibrils of Amyloid-beta and alpha-Synuclein, and Neuroprotection. *J Agric Food Chem* 64(41)**,** 7722-7732. doi: 10.1021/acs.jafc.6b03217.

Hou, Y., Wang, Y., Zhao, J., Li, X., Cui, J., Ding, J., et al. (2014). Smart Soup, a traditional Chinese medicine formula, ameliorates amyloid pathology and related cognitive deficits. *PLoS One* 9(11)**,** e111215. doi: 10.1371/journal.pone.0111215.

Hu, X., Qu, Y., Chu, Q., Li, W., and He, J. (2018). Investigation of the neuroprotective effects of Lycium barbarum water extract in apoptotic cells and Alzheimer's disease mice. *Mol Med Rep* 17(3)**,** 3599-3606. doi: 10.3892/mmr.2017.8310.

Hu, Z.; Huang,Y.; Liu, G.; Liu, F.; Zhou, W.; Zhang, Y., Analysis on the Rule of Using Chinese Herbal Compounds to Treat Alzheimer's Disease. Pharmacology and Clinics of Chinese Materia Medica 2012, 28, 252-256.

Huang, Y.C., Tsay, H.J., Lu, M.K., Lin, C.H., Yeh, C.W., Liu, H.K., et al. (2017). Astragalus membranaceus-Polysaccharides Ameliorates Obesity, Hepatic Steatosis, Neuroinflammation and Cognition Impairment without Affecting Amyloid Deposition in Metabolically Stressed APPswe/PS1dE9 Mice. *Int J Mol Sci* 18(12). doi: 10.3390/ijms18122746.

Hung, K.C., Huang, H.J., Wang, Y.T., and Lin, A.M. (2016). Baicalein attenuates alpha-synuclein aggregation, inflammasome activation and autophagy in the MPP(+)-treated nigrostriatal dopaminergic system in vivo. *J Ethnopharmacol* 194**,** 522-529. doi: 10.1016/j.jep.2016.10.040.

Huang, X.; Zhang, T.; Zhou, Y.; Zhou, B., Analysis of medication rules for the treatment of dementia based on the traditional Chinese medicine inheritance auxiliary system. Medical Diet and Health 2019, 96-97.

Iannuzzi, C., Borriello, M., Irace, G., Cammarota, M., Di Maro, A., and Sirangelo, I. (2017). Vanillin Affects Amyloid Aggregation and Non-Enzymatic Glycation in Human Insulin. *Sci Rep* 7(1)**,** 15086. doi: 10.1038/s41598-017-15503-5.

Ji, K., Zhao, Y., Yu, T., Wang, Z., Gong, H., Yang, X., et al. (2016). Inhibition effects of tanshinone on the aggregation of alpha-synuclein. *Food Funct* 7(1)**,** 409-416. doi: 10.1039/c5fo00664c.

Jia, S.L., Wu, X.L., Li, X.X., Dai, X.L., Gao, Z.L., Lu, Z., et al. (2016). Neuroprotective effects of liquiritin on cognitive deficits induced by soluble amyloid-beta1-42 oligomers injected into the hippocampus. *J Asian Nat Prod Res* 18(12)**,** 1186-1199. doi: 10.1080/10286020.2016.1201811.

Jiao, C., Gao, F., Ou, L., Yu, J., Li, M., Wei, P., et al. (2018). Tetrahydroxystilbene glycoside antagonizes beta-amyloid-induced inflammatory injury in microglia cells by regulating PU.1 expression. *Neuroreport* 29(10)**,** 787-793. doi: 10.1097/WNR.0000000000001032.

Jiao, Z.. Research on the Law of Syndrome and Treatment of Alzheimer's Disease Based on Data Mining. Doctor degree thesis, Liaoning University Of Traditional Chinese Medicine 2016.

Khan, H., Ullah, H., Aschner, M., Cheang, W.S., and Akkol, E.K. (2019). Neuroprotective Effects of Quercetin in Alzheimer's Disease. *Biomolecules* 10(1). doi: 10.3390/biom10010059.

Kim, H.J., Jung, S.W., Kim, S.Y., Cho, I.H., Kim, H.C., Rhim, H., et al. (2018). Panax ginseng as an adjuvant treatment for Alzheimer's disease. *J Ginseng Res* 42(4)**,** 401-411. doi: 10.1016/j.jgr.2017.12.008.

Korshavn, K.J., Jang, M., Kwak, Y.J., Kochi, A., Vertuani, S., Bhunia, A., et al. (2015). Reactivity of Metal-Free and Metal-Associated Amyloid-beta with Glycosylated Polyphenols and Their Esterified Derivatives. *Sci Rep* 5**,** 17842. doi: 10.1038/srep17842.

Kuboyama, T., Hirotsu, K., Arai, T., Yamasaki, H., and Tohda, C. (2017). Polygalae Radix Extract Prevents Axonal Degeneration and Memory Deficits in a Transgenic Mouse Model of Alzheimer's Disease. *Front Pharmacol* 8**,** 805. doi: 10.3389/fphar.2017.00805.

Lee, B., Shim, I., Lee, H., and Hahm, D.H. (2011). Rehmanniae Radix ameliorates scopolamine-induced learning and memory impairment in rats. *J Microbiol Biotechnol* 21(8)**,** 874-883. doi: 10.4014/jmb.1104.04012.

Lin, S.; Lu, J.; Song, K.; Chen, Z.; Jiang, S.; Pan, C.; Li, X.; Fu, X., Study on Compatibility of TCM Prescriptions for Treating Alzheimer Disease Based on Data Mining. Chin J Inf Tradit Chin Med 2015, 22, 41-44.

Ling, S., and Xu, J.W. (2016). Biological Activities of 2,3,5,4'-Tetrahydroxystilbene-2-O-beta-D-Glucoside in Antiaging and Antiaging-Related Disease Treatments. *Oxid Med Cell Longev* 2016**,** 4973239. doi: 10.1155/2016/4973239.

Link, P., Wetterauer, B., Fu, Y., and Wink, M. (2015). Extracts of Glycyrrhizae Radix et Rhizoma and isoliquiritigenin counteract amyloid-beta toxicity in Caenorhabditis elegans. *Planta Med* 81(5)**,** 357-362. doi: 10.1055/s-0035-1545724.

Liu, C., Chen, K., Lu, Y., Fang, Z., and Yu, G. (2018). Catalpol provides a protective effect on fibrillary Abeta1-42 -induced barrier disruption in an in vitro model of the blood-brain barrier. *Phytother Res* 32(6)**,** 1047-1055. doi: 10.1002/ptr.6043.

Lu, M.; Zhou, Y.; Li, X.; Sun, H.; Guo, J.; Wu, B.; Wu, M., Research on traditional Chinese medicine in treatment of Alzheimer's disease based on data mining. China Journal of Chinese Materia Medica 2020, 1-8.

May, B.H., Feng, M., Zhou, I.W., Chang, S.Y., Lu, S.C., Zhang, A.L., et al. (2016). Memory Impairment, Dementia, and Alzheimer's Disease in Classical and Contemporary Traditional Chinese Medicine. *J Altern Complement Med* 22(9)**,** 695-705. doi: 10.1089/acm.2016.0070.

Qi, Y., Cheng, X., Jing, H., Yan, T., Xiao, F., Wu, B., et al. (2019). Effect of Alpinia oxyphylla-Schisandra chinensis herb pair on inflammation and apoptosis in Alzheimer's disease mice model. *J Ethnopharmacol* 237**,** 28-38. doi: 10.1016/j.jep.2019.03.029.

Ou, F.. Data mining-based analysis on the regularity of ancient TCM prescription for amnestic mild cognitive impairment, Master degree thesis, Guangzhou University Of Traditional Chinese Medicine 2015.

Qu, Y., Zhang, Y., Pei, L., Wang, Y., Gao, L., Huang, Q., et al. (2011). New neuritogenic steroidal saponin from Ophiopogonis Radix (Thunb.) Ker-Gawl. *Biosci Biotechnol Biochem* 75(6)**,** 1201-1204. doi: 10.1271/bbb.110066.

Rajabian, A., Rameshrad, M., and Hosseinzadeh, H. (2019). Therapeutic potential of Panax ginseng and its constituents, ginsenosides and gintonin, in neurological and neurodegenerative disorders: a patent review. *Expert Opin Ther Pat* 29(1)**,** 55-72. doi: 10.1080/13543776.2019.1556258.

Rho, T., Choi, M.S., Jung, M., Kil, H.W., Hong, Y.D., and Yoon, K.D. (2019). Identification of fermented tea (Camellia sinensis) polyphenols and their inhibitory activities against amyloid-beta aggregation. *Phytochemistry* 160**,** 11-18. doi: 10.1016/j.phytochem.2018.12.013.

Ringman, J.M., Frautschy, S.A., Teng, E., Begum, A.N., Bardens, J., Beigi, M., et al. (2012). Oral curcumin for Alzheimer's disease: tolerability and efficacy in a 24-week randomized, double blind, placebo-controlled study. *Alzheimers Res Ther* 4(5)**,** 43. doi: 10.1186/alzrt146.

Savaskan, E., Mueller, H., Hoerr, R., von Gunten, A., and Gauthier, S. (2018). Treatment effects of Ginkgo biloba extract EGb 761(R) on the spectrum of behavioral and psychological symptoms of dementia: meta-analysis of randomized controlled trials. *Int Psychogeriatr* 30(3)**,** 285-293. doi: 10.1017/S1041610217001892.

Sgarbossa, A., Monti, S., Lenci, F., Bramanti, E., Bizzarri, R., and Barone, V. (2013). The effects of ferulic acid on beta-amyloid fibrillar structures investigated through experimental and computational techniques. *Biochim Biophys Acta* 1830(4)**,** 2924-2937. doi: 10.1016/j.bbagen.2012.12.023.

Tsunoda, T., Takase, M., and Shigemori, H. (2018). Structure-activity relationship of clovamide and its related compounds for the inhibition of amyloid beta aggregation. *Bioorg Med Chem* 26(12)**,** 3202-3209. doi: 10.1016/j.bmc.2018.04.044.

von Gunten, A., Schlaefke, S., and Uberla, K. (2016). Efficacy of Ginkgo biloba extract EGb 761((R)) in dementia with behavioural and psychological symptoms: A systematic review. *World J Biol Psychiatry* 17(8)**,** 622-633. doi: 10.3109/15622975.2015.1066513.

Wang, H.; Han, T.; Guo, W.; Zhang, T.; Jia, Y., Study on the medication rule of senile dementia based on cluster analysis. Journal of Shandong University of Traditional Chinese Medicine 2012, 36, 480-481+486.

Wang, L., Jin, G.F., Yu, H.H., Lu, X.H., Zou, Z.H., Liang, J.Q., et al. (2019). Protective effects of tenuifolin isolated from Polygalae Radix roots on neuronal apoptosis and learning and memory deficits in mice with Alzheimer's disease. *Food Funct* 10(11)**,** 7453-7460. doi: 10.1039/c9fo00994a.

Wang, L., Liu, S., Xu, J., Watanabe, N., Mayo, K.H., Li, J., et al. (2020). Emodin inhibits Abeta42 aggregation and improves cognitive deficits in Alzheimer's disease transgenic mice. *J Neurochem*. doi: 10.1111/jnc.15156.

Wang, X.. The study of prescription form and drug laws to treatment Alzheimer in Ming and Qing Dynasties. Master degree thesis, Xinjiang medical university 2010.

Wei, S.; Wang, Z.; Hu, S., Explore Regulatory in Prescription Compatibility of Chinese Medicine on Treatment of Alzheimer's Disease. *J Tradit Chin Med Univ Hunan* 2015, ***35***, 67-69.

Wu, J.Z., Ardah, M., Haikal, C., Svanbergsson, A., Diepenbroek, M., Vaikath, N.N., et al. (2019). Dihydromyricetin and Salvianolic acid B inhibit alpha-synuclein aggregation and enhance chaperone-mediated autophagy. *Transl Neurodegener* 8**,** 18. doi: 10.1186/s40035-019-0159-7.

Wu, W., Liang, X., Xie, G., Chen, L., Liu, W., Luo, G., et al. (2018). Synthesis and Evaluation of Novel Ligustrazine Derivatives as Multi-Targeted Inhibitors for the Treatment of Alzheimer's Disease. *Molecules* 23(10). doi: 10.3390/molecules23102540.

Xie, H., Wang, J.R., Yau, L.F., Liu, Y., Liu, L., Han, Q.B., et al. (2014). Catechins and procyanidins of Ginkgo biloba show potent activities towards the inhibition of beta-amyloid peptide aggregation and destabilization of preformed fibrils. *Molecules* 19(4)**,** 5119-5134. doi: 10.3390/molecules19045119.

Xia, X.. Analysis of the prescription rule based on the traditional Chinese medicine inheritance auxiliary system for the treatment of senile cognitive impairment, Master degree thesis, China Academy of Chinese Medical Sciences 2015.

Yan, J., Study on the medication rule of the literature on the treatment of senile dementia with traditional Chinese medicine, Master degree thesis, Hubei University Of Traditional Chinese Medicine 2007.

Yang, C., Bao, X., Zhang, L., Li, Y., Li, L., and Zhang, L. (2020). Cornel iridoid glycoside ameliorates cognitive deficits in APP/PS1/tau triple transgenic mice by attenuating amyloid-beta, tau hyperphosphorylation and neurotrophic dysfunction. *Ann Transl Med* 8(6)**,** 328. doi: 10.21037/atm.2020.02.138.

Yang, Y., Xuan, L., Chen, H., Dai, S., Ji, L., Bao, Y., et al. (2017). Neuroprotective Effects and Mechanism of beta-Asarone against Abeta1-42-Induced Injury in Astrocytes. *Evid Based Complement Alternat Med* 2017**,** 8516518. doi: 10.1155/2017/8516518.

Yi, Y.; Fang, R.; Ge, J.; Cheng, S.; Wang, G.; Liu, L., Analysis on medication rules for treatment of dementia by ancient physicians based on data mining methods. China Journal of Chinese Materia Medica 2018, 43, 3376-3381.

Yu, M., Chen, X., Liu, J., Ma, Q., Zhuo, Z., Chen, H., et al. (2019). Gallic acid disruption of Abeta1-42 aggregation rescues cognitive decline of APP/PS1 double transgenic mouse. *Neurobiol Dis* 124**,** 67-80. doi: 10.1016/j.nbd.2018.11.009.

Zeng, Y.; Zhu, W.; Wen, Y.; Zhou, X.; Xie, M., Research on Experienced Doctors’Treatment of Senile Dementia Based on Association Rule. Chin J Inf Tradit Chin Med 2015, 22, 31-33.

Zhang, W., Zhi, D., Ren, H., Wang, D., Wang, X., Zhang, Z., et al. (2016a). Shengmai Formula Ameliorates Pathological Characteristics in AD C. elegans. *Cell Mol Neurobiol* 36(8)**,** 1291-1302. doi: 10.1007/s10571-015-0326-z.

Zhang, X.Z., Qian, S.S., Zhang, Y.J., and Wang, R.Q. (2016b). Salvia miltiorrhiza: A source for anti-Alzheimer's disease drugs. *Pharm Biol* 54(1)**,** 18-24. doi: 10.3109/13880209.2015.1027408.

Zhang, Y., Kong, W.N., and Chai, X.Q. (2018). Compound of icariin, astragalus, and puerarin mitigates iron overload in the cerebral cortex of Alzheimer's disease mice. *Neural Regen Res* 13(4)**,** 731-736. doi: 10.4103/1673-5374.230302.

Zhao, L.H., Ding, Y.X., Zhang, L., and Li, L. (2010). Cornel iridoid glycoside improves memory ability and promotes neuronal survival in fimbria-fornix transected rats. *Eur J Pharmacol* 647(1-3)**,** 68-74. doi: 10.1016/j.ejphar.2010.08.016.

Zheng, T., Jiang, H., Jin, R., Zhao, Y., Bai, Y., Xu, H., et al. (2019). Ginsenoside Rg1 attenuates protein aggregation and inflammatory response following cerebral ischemia and reperfusion injury. *Eur J Pharmacol* 853**,** 65-73. doi: 10.1016/j.ejphar.2019.02.018.

Zhou, L., Liao, W., Chen, X., Yue, H., Li, S., and Ding, K. (2018). An arabinogalactan from fruits of Lycium barbarum L. inhibits production and aggregation of Abeta42. *Carbohydr Polym* 195**,** 643-651. doi: 10.1016/j.carbpol.2018.05.022.

Zhou, L.; Zhang, W.; Zeng, L.; Jia, B., Study on the rule of compound prescription for senile dementia. *Liaoning Journal of Traditional Chinese Medicine* 2005, 243-244.

Zhu, B., Zhang, Q.L., Hua, J.W., Cheng, W.L., and Qin, L.P. (2018). The traditional uses, phytochemistry, and pharmacology of Atractylodis Macrocephalae Rhizoma: A review. *J Ethnopharmacol* 226**,** 143-167. doi: 10.1016/j.jep.2018.08.023.

Zhu, X.. Study on the Regularity of Alzheimer 's Disease in Traditional Chinese Medicine Based on Association Rules, Master degree thesis, Shandong University Of Traditional Chinese Medicine 2017.

Zong, X.; Ji, X.; Wei, F.; Shi, Z., Analysis on prescription rules of treating senile dementia based on traditional Chinese medicine inheritance auxiliary systems. China Journal of Chinese Materia Medica 2014, 39, 640-643.
